# Obstructive sleep apnea detection during wakefulness: a comprehensive methodological review

**DOI:** 10.1007/s11517-024-03020-3

**Published:** 2024-01-27

**Authors:** Ali Mohammad Alqudah, Ahmed Elwali, Brendan Kupiak, Farahnaz Hajipour, Natasha Jacobson, Zahra Moussavi

**Affiliations:** 1 Biomedical Engineering Program, University of Manitoba, 66 Chancellors Cir, Winnipeg, MB R3T 2N2 Canada; 2 Biomedical Engineering Program, Marian University, 3200 Cold Sprint Road, Indianapolis, IN 46222-1997 USA; 3 Electrical and Computer Engineering Department, University of Manitoba, 66 Chancellors Cir, Winnipeg, MB R3T 2N2 Canada; 4 SkipTheDishes, Winnipeg, Canada; 5 Biosystems Engineering Department, University of Manitoba, 66 Chancellors Cir, Winnipeg, MB R3T 2N2 Canada

**Keywords:** Sleep apnea, Classification, Home sleep study, OSA, Polysomnography, Questionnaire, Screening wakefulness

## Abstract

Obstructive sleep apnea (OSA) is a chronic condition affecting up to 1 billion people, globally. Despite this spread, OSA is still thought to be underdiagnosed. Lack of diagnosis is largely attributed to the high cost, resource-intensive, and time-consuming nature of existing diagnostic technologies during sleep. As individuals with OSA do not show many symptoms other than daytime sleepiness, predicting OSA while the individual is awake (wakefulness) is quite challenging. However, research especially in the last decade has shown promising results for quick and accurate methodologies to predict OSA during wakefulness. Furthermore, advances in machine learning algorithms offer new ways to analyze the measured data with more precision. With a widening research outlook, the present review compares methodologies for OSA screening during wakefulness, and recommendations are made for avenues of future research and study designs.

**Graphical abstract:**

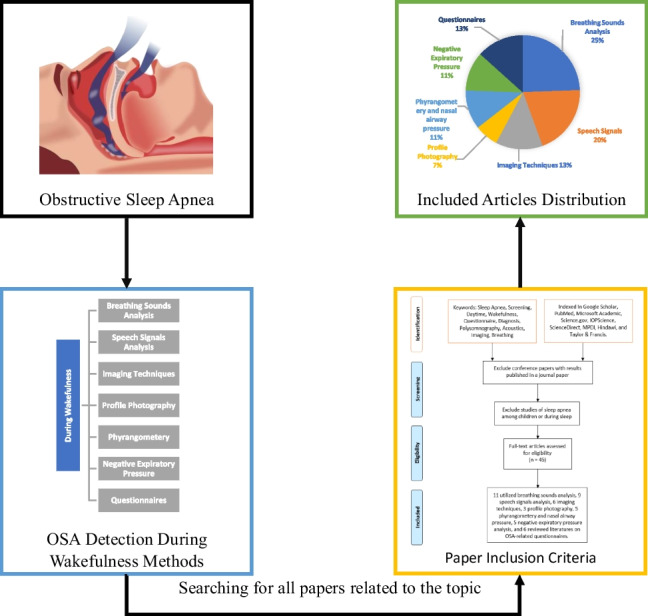

## Introduction

Sleep apnea is a prevalent respiratory disorder defined as periods of airflow cessation (apnea) or reduced airflow by more than 30% (hypopnea) associated with at least a 3% drop in blood oxygen saturation level (SpO2) [1, 2]. Sleep apnea is differentiated into three types: obstructive, central, and mixed apnea. Obstructive sleep apnea (OSA) is the most common type, accounting for more than 75% of cases. In contrast, central sleep apnea is relatively rare (about 5% of cases), with 20% of cases presenting with a mix of central and OSA [1, 2]. OSA is characterized by repeated [3] episodes of complete (apnea) and partial (hypopnea) cessation of breathing due to pharyngeal collapse during sleep [3]. OSA severity is measured by the apnea/hypopnea index (AHI). AHI is the number of apneic and hypopneic events per hour of sleep. To constitute an event, apnea/hypopnea should last at least 10 s and be associated with more than a 3% drop in blood oxygen saturation [4]. An OSA event may be accompanied by an increased heart rate and blood pressure, as well as subsequent arousals from sleep to restore the upper airway (UA) patency [3]. Signs and symptoms of OSA include daytime sleepiness, tiredness, depression, morning headaches, nighttime gasping and choking, and snoring. While it is debatable whether OSA causes other health issues, OSA has been linked with cardiovascular and cerebrovascular disease [5–8], reduced attention, an increased risk for car accidents [9], perioperative morbidity, and post-surgery mortality [10]. Commonly, AHI ratings between 0–5, 5–15, 15–30, and > 30 are referred to as non-, mild-, moderate-, and severe-OSA, respectively [10].

### Brief history of sleep apnea research during wakefulness

Research suggests that a significant percentage of the OSA population (> 80%) remains undiagnosed and untreated [10, 11]. In the USA, the direct and indirect costs associated with untreated OSA are estimated to be between $65 and $165 billion, annually [12]. Under-diagnosis and, in turn, treatment delays for severe cases of OSA may further increase indirect costs while increasing the risk of associated vehicle accidents [13]. The main reasons for OSA under-diagnosis are the limited resources in healthcare (i.e., sleep laboratories, sleep technicians) and the inefficiency of existing diagnostic tooling. The gold standard diagnostic assessment of OSA is an overnight full polysomnography (PSG), in which more than 15 biological signals such as brain waves, respiratory flow, blood oxygen saturation, heart and muscle signals, and snoring sounds are recorded and monitored by a certified sleep technician at a sleep center. Though PSG remains the most accurate OSA diagnostic test, it is an expensive, time-consuming, and labor-intensive procedure. As a result, there is typically a long waiting list (occasionally up to 1 year [14]) to conduct an overnight PSG study.

The second-best diagnostic assessment is the home sleep test (HST): a portable, simplified version of the PSG device that allows patients to self-monitor overnight at home [14] although the diagnosis is being made by a specialist after screening data of the HST [14]. The HST records a significantly smaller number of signals (3–4) than PSG and is less precise as it relies on the patient to perform the recording themselves. A clinically approved HST device records at least three main signals: nasal airflow, EEG to detect sleep stage, and blood oxygen saturation. Beyond PSG and HST studies, there also exist several overnight acoustic sleep studies that use tracheal breathing sounds in addition to blood oxygenation; they have resulted in high accuracy (> 96%) compared to gold standard PSG [15].

Given the significant time and labor-intensive demand of PSG and HST, daytime testing (during *wakefulness*) to detect OSA is a very desirable alternative. During wakefulness, OSA is clinically assessed by questionnaires such as STOP-BANG [16] or the Epworth Sleepiness Score [17]. Despite the simplicity of the questionnaires over traditional overnight testing, OSA risk estimation by such questionnaires has very low specificity around 25%, thus, implying a very high false-positive rate [18, 19]. However, many clinicians, particularly anesthesiologists, continue to use quick OSA assessment questionnaires as OSA is a major risk factor for complications after general anesthesia [20].

The history of OSA during wakefulness using other methods than the abovementioned questionnaires has been intricately tied to the exploration of anatomical structures and physiological changes, primarily through the lens of advanced imaging studies [21]. Hypotheses initially centered on the role of upper airway collapsibility and the anatomical predispositions contributing to its occurrence during wakefulness. With the advent of imaging techniques such as magnetic resonance imaging (MRI) and computed tomography (CT), researchers gained the ability to visualize and analyze the upper airway’s structural complexities in both OSA and non-OSA subjects [22]. These imaging studies have provided insights into the differences in the upper airway dimensions, soft tissue characteristics, and anatomical variations between OSA and non-OSA groups, offering critical clues to the pathophysiology of OSA during wakefulness. Subsequent investigations have continued to refine our understanding of the intricate interplay between anatomical predispositions and the development of OSA, fostering advancements in diagnostic approaches and therapeutic interventions for this complex sleep-related disorder [21, 22]. Thus, several research groups have attempted to analyze breathing or vocal sounds recorded in a few minutes during *wakefulness* to estimate OSA severity based on the anatomical differences’ effects on these sounds.

**Fig. 1:**
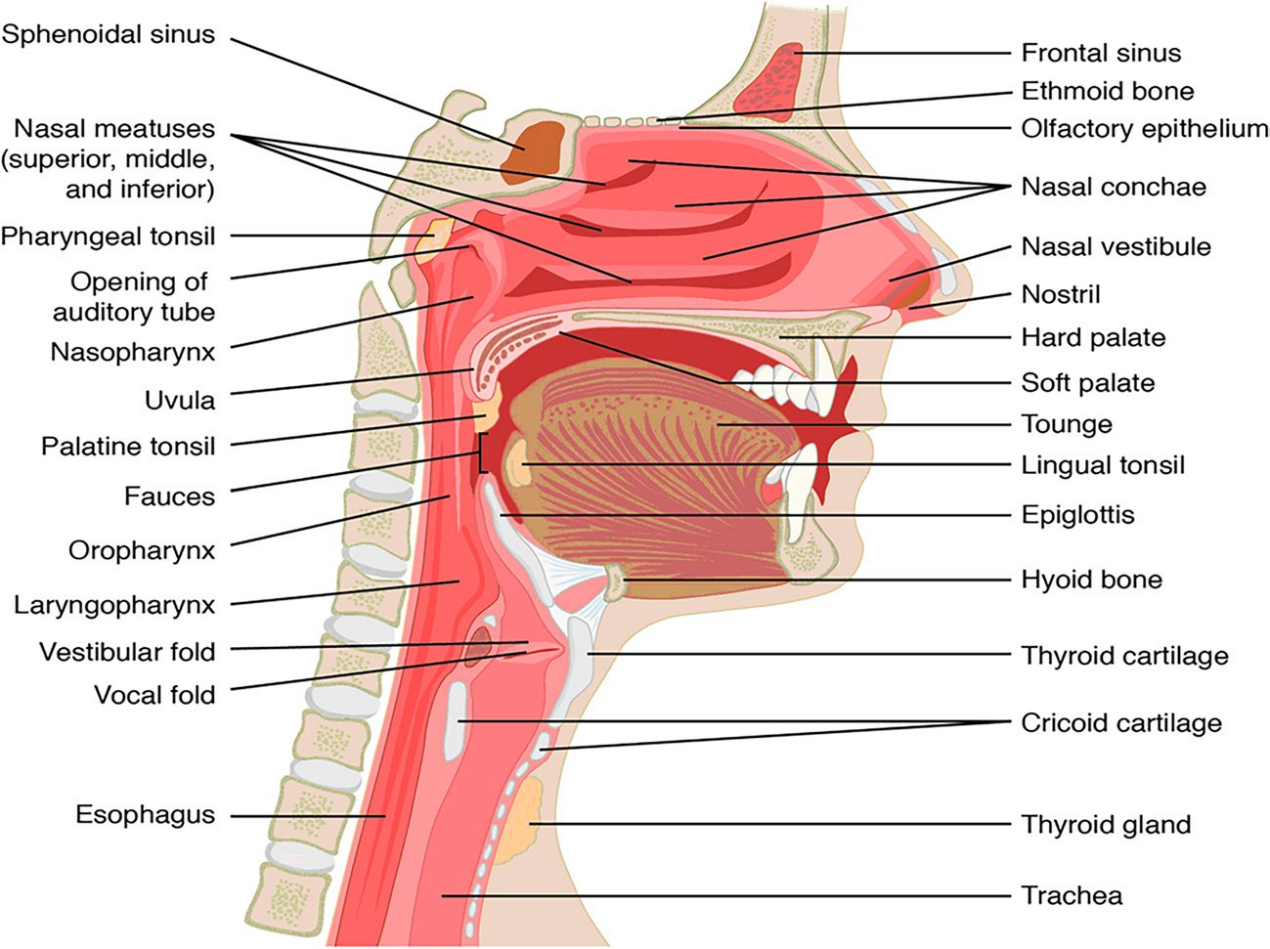
Anatomy of the upper airway [26] (CC BY 4.0)

Contrary to OSA detection using breathing sounds during sleep, the challenge of wakeful OSA detection is the lack of noticeable breathing difficulty, even during exercise, while an individual is awake [23]. This is most likely attributed, in general, to an increase in the UA dilator muscle’s activity (especially for the genioglossus muscle) during wakefulness, which compensates for the physiological UA changes due to OSA [22–24]. Nevertheless, imaging studies during wakefulness have confirmed the existence of morphological and mechanical differences in individuals with various OSA severities [21, 22, 24]. The sleep-related narrowing and increased compliance or collapsibility of the UA are critical contributors to the pathogenesis of OSA [25]. Further, compared to their healthy counterparts, individuals with OSA have been shown to have an increased pharyngeal length, a thick posterior, a long and thick soft palate, and a more compliant airway [22, 24]. Overall, OSA individuals tend to have a circular velopharynx shape rather than an elliptic shape with the long axis oriented in the lateral plane as in non-OSA individuals [21]. To navigate the morphologic changes of OSA, the UA anatomy is illustrated in Fig. 1.

As mentioned above, despite these morphological and structural changes in the UA, OSA individuals do not experience any breathing difficulty while awake. However, given that breathing and vocal sounds are generated by the flow of air in the UA, it has been hypothesized [27] that the breathing sounds and vocal sounds reflect UA abnormalities during wakefulness. As such, OSA prediction during [26–31] wakefulness has received significant attention in recent years, leading to the development of seven notable OSA detection technologies: (1) imaging techniques, (2) negative expiratory pressure, (3) facial image landmarks, (4) acoustic pharyngometer and nasal airway pressure, (5) breathing sound analysis, (6) speech signal analysis, and (7) questionnaires.

### Objective

With the increasing demand for efficient and accurate OSA screening tools during wakefulness, it is the objective of the present manuscript to review existing OSA detection technologies during wakefulness and suggest avenues for future research. A list of acronyms used throughout this paper is presented in Table 1.

**Table 1:** List of abbreviations used in this paper in alphabetical order

Term	Definition	Term	Definition
AHI	Apnea/hypopnea index	MRI	Magnetic resonance imaging
AUC	Area under curve	NEP	Negative expiratory pressure
BMI	Body mass index	ORP	Odds ratio products
CNN	Convolution neural network	OSA	Obstructive sleep apnea
CT	Computed tomography	PSG	Polysomnography
CPAP	Continuous positive airway pressure	QI	Quantitative index
EEG	Electroencephalography	RF	Random forest
ESS	Epworth Sleepiness Scale	ROC	Receiver operating characteristics
FVC	Flow-volume curve	REM	Rapid eye movement
GA	Genetic algorithm	SDB	Sleep-disordered breathing
GMM	Gaussian mixture model	SpO2	Mean oxygen saturation
HST	Home sleep test	SVM	Support vector machine
LASSO	Least absolute shrinkage and selection operator	SVR	Support vector regression
LDA	Linear discriminant analysis	TSI	Tracheal sound intensity
LR	Logistic regression	UA	Upper airway
MFCCS	Mel Frequency Cepstral Coefficients	UARS	Upper airway resistance syndrome
MLP	Multi-layer perceptron		

## Methods

**Fig. 2:**
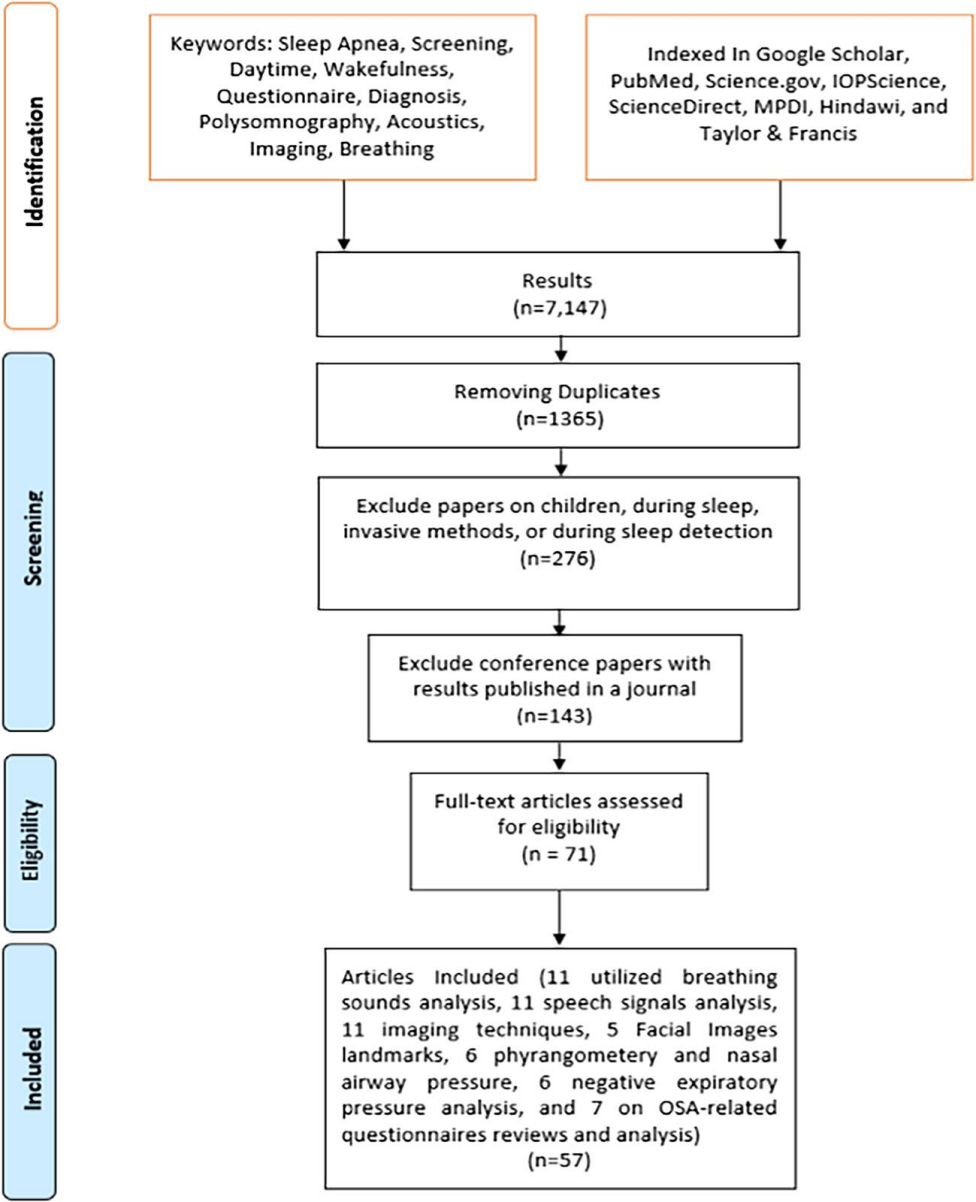
Flow diagram of paper inclusion and exclusion

We included all journal papers related to OSA screening in adults during wakefulness; among conference papers, only those with results not published in a journal paper were included. The search for relevant papers in this review encompassed the period from 1980 to 2023. Relevant manuscripts were identified by searching Google Scholar, PubMed, Science.gov, IOPscience, ScienceDirect, MPDI, Hindawi, and Taylor & Francis. The collection of papers was performed by four researchers (co-authors) through an online shared folder. The search strategy involved the utilization of combined keywords related to sleep apnea and various aspects of screening, daytime symptoms, wakefulness, and specific diagnostic techniques such as polysomnography, acoustics, imaging, and breathing. Figure 2 shows the flow diagram of the searching algorithm. Notably, the search approach emphasized the combination of keywords rather than their isolated use to ensure a comprehensive and targeted search process. The search was performed using a combination of the following keywords: sleep apnea, screening, daytime, wakefulness, questionnaire, diagnosis, polysomnography, acoustics, imaging, and breathing. A sample of queries used during collecting papers is sleep apnea AND screening AND diagnosis, sleep apnea AND wakefulness AND acoustics, sleep apnea AND wakefulness AND breathing, and sleep apnea AND wakefulness AND imaging.

The assessment of search outcomes was broadened, ceasing when no more pertinent results were evident, with the recognition that significant findings were usually discovered within the initial pages of the search results. The inclusion of papers in the preliminary set was based on a hierarchical assessment, beginning with an examination of titles indicating potential relevance and further scrutiny of corresponding abstracts, which resulted in an initial set of 7147 papers reduced to 1365 after removing duplications. The selection process of the papers involved a meticulous review by at least two co-authors for each technology and ensuring the inclusion of high-quality and pertinent research. The following exclusion criteria were applied:

1. Exclusion of sleep apnea studies in children because OSA pathology is different in adults and children.
2. Exclusion of studies during sleep since the focus of this paper is detection during wakefulness.
3. Exclusion of studies using invasive techniques because the focus of the paper is on non-invasive methods.

**Fig. 3:**
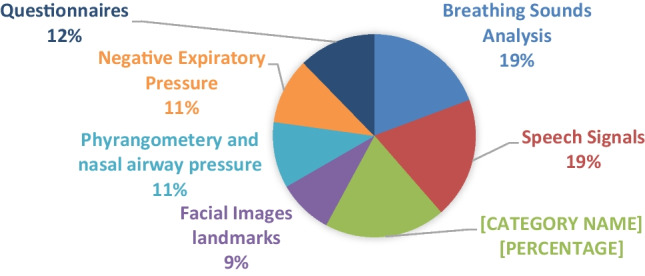
Distribution of articles over topics in this review

After applying the above exclusion criteria, the number of included papers was reduced to 143. From those, 71 full-text articles were included after applying other exclusion criteria such as removing those with incomplete or missing data, lack of full-text access, or publication in a language other than English. The remaining articles were then passed through an assessment process to focus solely on OSA detection during wakefulness without any analysis during sleep, except for polysomnography (PSG) for the apnea–hypopnea index (AHI), which is a calculation often used for accuracy measure of the algorithms. As a result, 57 papers were selected for review in this manuscript. These papers highlight various techniques such as breathing sound analysis (*n* = 11), speech signal analysis (*n* = 11), imaging techniques (*n* = 11), facial image landmarks (*n* = 5), pharyngometry and nasal airway pressure (*n* = 6), negative expiratory pressure analysis (*n* = 6), and analysis of OSA-related questionnaires (*n* = 7). The outcomes of the techniques used in each category are presented separately, followed by a general discussion. Figure 3 shows the distribution of articles over topics selected in this paper.

**Fig. 4:**
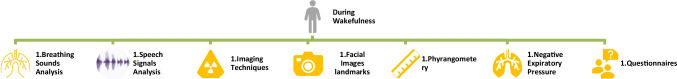
OSA detection during wakefulness technologies

It should be noted that not all the studies reviewed in this manuscript intended to detect OSA; for example, the imaging studies only investigated the UA physiological and structural changes during wakefulness without the intention of imaging to develop a screening tool for OSA detection. Regardless, these imaging studies are reviewed here because their findings support further research on OSA screening technologies during wakefulness. Since the imaging studies’ findings have provided the main rationale for OSA screening technologies during wakefulness, we present those findings first and then present existing technologies for OSA. Figure 4 shows a graphical summary of the techniques used for OSA detection during wakefulness that are covered and discussed in this paper.

In this review, the reason for not employing the PRISMA guidelines is mainly due to our focus on OSA non-invasive screening techniques during wakefulness in adults. The inclusion criteria were designed to include both journal and conference papers with unpublished results in the journal, while exclusion criteria were designed to exclude studies that involved children, invasive techniques, and sleep analysis. The developed search methodology utilized a combination of keywords which were different from the strict PRISMA, ensuring that the outcomes aligned with the study’s objectives and maintained methodological transparency.

### Imaging

Different medical imaging techniques have long been key to providing insights into the deep anatomical and functional information about human organs, and through them, researchers can track changes in an organ’s size, shape, or dimensions. Several imaging modalities such as cephalometric X-rays, computed tomography (CT), ultrasound, magnetic resonance imaging (MRI), and endoscopy have been used to investigate the UA structural and morphological changes in patients with OSA.

For diagnostic purposes using X-ray cephalometric, different physiological aspects and parameters may be measured via screening the mandibular deficiencies [28]. Commonly, cephalometric analysis is performed by measuring the angles and distances between various cephalometric landmarks, and it is very useful for detecting anatomical changes due to OSA in an individual, but those changes are not enough to identify OSA accurately [28]. Nevertheless, imaging studies using X-rays on 15 participants have found the length of the soft palate to be longer, and position of the hyoid bone to be inferior, and a narrower posterior airway compared to those in healthy individuals [28].

Unlike X-rays, CT scans provide axial images, and volumetric scans and measurements instead of planar images. Different studies have used either traditional CT scans [29] or Cine CT (high-speed CT imaging) to study the UA changes due to OSA [30]. These studies explored the changes in the UA anatomy during wakeful and tidal breathing, and the presence of a sawtooth pattern in the flow-volume curves due to OSA [29] and the pharyngeal area during a wakeful respiratory cycle rack the respiratory cycle [30]. The results of the study in [29] showed that there is a strong relationship between pharyngeal area and OSA, where the mean area of the nasopharynx, oropharynx (the most severely narrowed part), and hypopharynx was significantly less in OSA individuals than those in healthy controls. Moreover, those regional measurements correlated with AHI and blood oxygen saturation levels without correlating with age or obesity. Also, the results, congruent with those findings reported in [28], showed no definitive evidence relating a sawtooth pattern in FVC and OSA. Also congruent with the outcomes of the study in [28], the results of [29] showed that low retropalatal region presented the greatest difference due to OSA, and the median minimal airway size was smaller in snorers than that in controls. However, that difference was not statistically significant and was also found to be correlated with body mass index (BMI), neck circumference, AHI, and blood oxygen saturation level. Furthermore, it was noticed that the OSA patients’ UA had overall larger dimensional changes during the respiratory cycle than that of both the snorer and control groups, thus, indicating a more distensible UA in OSA individuals than that in healthy ones. Finally, as expected, most of the dimensional changes occurred during expiration, during which the airway expanded greatly and then collapsed [28].

Lately, MRI scans have been used to study the UA changes in OSA subjects. Compared to CT scans, MRI provides higher resolution and more details about the anatomical structures in the human especially the soft tissues. Also, like CT, MRI can provide volumetric images and measurements but with higher resolution. An imaging study was conducted during sleep as well as wakefulness using ultrafast MRI (one image per 0.8 s); the study aimed to examine the UA structure between 17 OSA individuals and their age-matched 8 healthy controls [31]. The major finding of the study was that the velopharynx of apneic individuals was smaller than that of healthy ones during the respiratory cycle. The variation of the velopharynx area was greater in apneic patients, particularly during sleep. The authors suggested this difference could be due to the increased compliance of the velopharynx in apneic individuals. Additionally, the apneic individuals during sleep showed a more circular velopharynx. Overall, it was concluded that changes in the velopharynx area and diameter during the respiratory cycle were greater in apneic individuals than those in healthy controls; this trend was more pronounced during sleep [31].

Another research examined the prognostic value of the lateral pharyngeal wall’ (LPW) thickness for the detection of OSA using two both US and MRI [32]. One hundred individuals with and without OSA (36 healthy controls and 64 OSA subjects) were enrolled in the study and performed an overnight PSG, and then the LPW thickness was measured using 1.5-T MRI and ultrasound during wakefulness. The ultrasound assessment was conducted during rest and Müller’s maneuver. The MRI results showed a significantly greater LPW thickness in the OSA group, while the ultrasound results showed a significant difference between the two groups only during the left side with Müller’s maneuver. Also, in general, a significant correlation was observed between LPW thickness and BMI, where patients with high BMI showed higher LPW thickness. Moreover, in terms of sex, males showed higher LPW thickness than females either in MRI or ultrasound assessment using left-sided Müller’s maneuver. Overall, AHI was correlated with LPW thickness, and the obstruction severity of LPW was correlated with LPW thickness; in addition, the LPW collapse was significantly correlated with AHI. Finally, 93% effectiveness in OSA prognostication was achieved using anthropometric data and the LPW thickness measured by ultrasound and 89% effectiveness using only LPW thickness. Moreover, using the MRI for detecting OSA during wakefulness and LPW-based obstruction resulted in 90% and 84% accuracy, respectively. Ultrasound data analysis successfully detected LPW-based collapse severity in 67% of cases.

- Another study focused on comparing the findings of the drug-induced sleep endoscopy (DISE) with the modified Mallampati score and Müller’s maneuver evaluation [33]. The comparison was done based on nose–oropharynx–hypopharynx–larynx (NOHL) for 43 individuals with moderate to severe OSA. The results showed the degree of collapsibility was significant only at the hypopharyngeal level, where 41.8% of the individuals during wakefulness and 88.3% in DISE (*p* < 0.0001) showed a hypopharyngeal obstruction. Moreover, 18.6% and 4.6% of the subjects showed laryngeal obstruction during wakefulness and DISE examination, respectively. However, the DISE succeeded in identifying the incidence of multilevel collapses (*p* = 0.001), while the incidence of oropharyngeal obstruction in patients with Mallampati scores I and II was significantly higher in DISE compared to that measured by Müller’s maneuver (*p* = 0.021).
- Some other studies have focused on the ability to use ultrasound or MRI imaging of the tongue for OSA prediction [32, 34]. One research [34] showed the ability of OSA prediction using AI applied to ultrasound imaging of the UA and subcutaneous adipose tissues (SAT) in the regions of the neck, chest, and abdomen measurements [35]. The data was collected from 100 individuals, 36 without OSA and 64 with different OSA severity (32 mild, 32 moderately severe) based on their overnight PSG scores, while the DISE was used to determine the obstruction location and configuration. The results showed using the SAT ultrasound and anthropometric data; the oropharyngeal and tongue-based obstructions could be predicted with 64% and 72% accuracies, respectively. In oropharyngeal obstruction prediction, the most important features were found to be BMI, abdominal and hip circumferences, and submental SAT and SAT above the second intercostal space on the left. Furthermore, for tongue-based obstruction, the most important features were found to be height; SAT measured 2 cm above the umbilicus and submental SAT. Overall, the OSA prediction using the parameters mentioned above had a sensitivity of 100% and a specificity of 91.7%. The second research [34] extracted geometrical parameters of the tongue from ultrasound and MRI images in OSA subjects based on sex, age, and BMI among 100 individuals (64 with OSA and AHI ≥ 5). The quadratic discriminant analysis was performed, and the results showed males compared to females had higher tongue volumes and axial diameter during Müller’s maneuver of ultrasound and coronal diameter of the MRI. All the examined MRI parameters were found significantly correlated with AHI among females with OSA; also, BMI showed a stronger correlation with AHI in females than in males. Using tongue parameters and anthropometric values, ultrasound analysis showed a sensitivity of 94% and a specificity of 98%, while MRI analysis showed 56% sensitivity and 92% specificity. Another study used MRI to analyze upper airway changes during tidal breathing in the OSA group and healthy controls [36]. The study used dynamic MRI where subjects were free to breathe during acquisition. While overall structure differences were minimal, OSA subjects had a narrower airway at a specific level. Significant variations in the upper airway size change over tidal breathing were observed in the OSA group, particularly in the low retropalatal/high retroglossal region during wakefulness and sleep. In OSA subjects, the collapsed airway during sleep aligned with the region showing the greatest changes in caliber while awake with tidal breathing. These results suggest a potential application for dynamic OSA imaging during wakefulness.
- In the surveyed literature, three studies used MRI imaging to observe the UA regions during wakefulness [31, 32, 36, 37]. The findings in [36] were consistent with those using CT imaging [29, 30]. The results showed narrower low retropalatal/high retroglossal regions in OSA individuals in comparison to those in healthy controls. Greater changes in the UA during respiration were observed, while they were also seen in the low retropalatal/high retroglossal regions during both sleep and wakefulness. Unlike the conventional CT imaging study [30], the subjects of this study [36] were free to breathe through their nose or mouth. Additionally, the study found that the differences in the UA area between the OSA and non-OSA groups could be completely attributed to differences in the anterior–posterior diameter as there was no significant difference in the lateral diameter between the groups. Thereafter, the finding by [37] using volumetric MRI showed that the volumes of all measured soft tissues were significantly greater in OSA subjects than those in controls, and each of these volumes was associated with an increased risk of OSA according to odds ratio products (ORP) [38]. Moreover, the study in [37] found total tongue and total lateral wall volumes were significant independent risk factors of OSA. Also, the average area and minimum area of the retropalatal region [36] were found significantly smaller in OSA subjects [37]. Contrary to previous work [36], both the lateral and anterior–posterior diameters were found smaller in OSA subjects in [37]; this could be due to the small sample size in the study reported in [36], noting that, although the difference in lateral diameter was significant, it was smaller than the difference in anterior–posterior diameter. Furthermore, 2D soft tissue measurements showed that the lateral pharyngeal wall was larger in OSA subjects and was associated with an increased risk of OSA [32]; this is congruent with the findings in [32] using 1.5-T MRI that showed larger LPW thickness in OSA group, while the associated risk of the volumetric measurements was substantially greater [32].

**Fig. 5:**
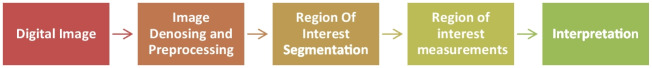
Detailed process of medical image analysis

While ultrasound has the worst resolution compared to MRI and CT, some researchers have shown that using only ultrasound image analysis has the potential to identify OSA individuals effectively [32–34, 39]. The imaging technologies (excluding ultrasounds) discussed above provide valuable insights into the anatomic and physiological changes of the UA due to OSA and provide evidence for various risk factors of OSA. However, the main drawback of these technologies is their high cost and their invasive nature (i.e., exposure to radiation or magnetic fields lead); although they are considered minimally invasive, they are not considered a necessary diagnostic tool for OSA. Moreover, medical images are passed through a series of processing techniques before starting the analysis [39]. Figure 5 shows the process sequence for the analysis of medical images. As a result, the sample size in imaging studies has been limited. That said, the studies presented have shown that the UA of OSA patients exhibits many of the same properties during sleep and wakefulness. As such, there is significant research interest in potential OSA screening tools exploiting the anatomic and physiological indicators found in imaging during wakefulness and making it a feasible option. Table 2 presents a summary of all investigated papers in this section.

**Table 2:** Summary of key findings of the investigated papers for medical imaging technique

Paper	Technique	Sample size	Key result summary
[40]	Ultrasound	21 with AHI < 15 and 20 with AHI > 15	A significant correlation between the length of the tongue base and AHI even after controlling for BMI. Training sensitivity and specificity of 80% and 67%
[28]	X-ray scan and flow-volume curve	10 with AHI > 10 and 5 healthy controls for X-ray and 12 with AHI > 10 and 5 healthy controls for flow-volume loop	OSA is characterized by mandibular deficiency, narrow posterior airway, long soft palate, and inferiorly positioned hyoid bone
[29]	CT scan and flow-volume curve	20 with AHI > 5 and 10 healthy controls	OSA was characterized by narrow nasopharynx, oropharynx, and hypopharynx and identified sawtooth patterns in flow-volume curves
[30]	CT scan and flow meter	15 with AHI < 2, 14 snorers/mild with 2 < AHI < 15 and 13 with AHI > 15	Moderate/severe OSA is characterized by a big change in the transition between inspiration and expiration and vice versa, constant cross-sectional area during inspiration, and a high overall percentage change in UA size during respiration
[37]	Volumetric MRI	48 with AHI < 5 and 48 with AHI ≥ 15	OSA is characterized by a smaller retropalatal region, larger tongue, soft palate, lateral walls and total soft tissues, and thicker lateral pharyngeal wall
[36]	MRI, electroencephalography (EEG), and nasal/oral flow	7 with AHI ≥ 15 and 7 BMI matched with AHI < 5	OSA is characterized by smaller UA, especially in the low-retropalatal/high-retroglossal region
[31]	Ultra-fast MRI	17 OSA subjects with AHI ≥ 10 and 8 control Subjects	OSA subjects have a smaller area of the velopharynx during part of the respiratory cycle. This variation was greater in apneic patients than in controls, particularly during sleep
[32]	Ultrasound and MRI	36 non-OSA subjects and 64 OSA subjects	The OSA group significantly has greater LPW thickness using MRI, while the significant difference between the two groups appears in the ultrasound only during left side MM. MRI for detecting OSA results in 90% and LPW-based obstruction in 84%
[33]	Endoscopy	43 subjects with AHI ≥ 15	The collapsibility was significant only at the hypopharyngeal level (41.8% during wakefulness and 88.3% in DISE). 18.6% and 4.6% of patients showed laryngeal obstruction during awake and DISE examinations, respectively
[35]	Ultrasound	36 with AHI < 5, 32 with 5 ≤ AHI < 15 and 32 with AHI ≥ 15	OSA prediction using the ultrasound parameters achieved a sensitivity of 100% and a specificity of 91.7%
[34]	Ultrasound and MRI	36 with AHI < 5 and 64 with AHI ≥ 5	Using tongue parameters and anthropometric values, an ultrasound sensitivity of 94% and a specificity of 98% were achieved while MRI has 56% sensitivity and 92% specificity

In summary, medical imaging techniques play a critical role in the detection and analysis of OSA detection and provide insights into the intricate anatomical and physiological changes associated with the condition that are used to form the hypothesis for other methods. Moreover, highlights the varying capabilities of each imaging modality in delineating the characteristics of OSA. Cephalometric X-rays, for instance, are valuable in detecting specific mandibular deficiencies, yet their ability to provide conclusive evidence for OSA diagnosis appears limited. In contrast, CT scans offer detailed axial and volumetric images, revealing strong correlations between pharyngeal area and OSA, demonstrating their potential for precise anatomical analysis. MRI, with its high-resolution imaging of soft tissues, emphasizes significant differences in the velopharynx area and diameter between OSA patients and healthy individuals during the respiratory cycle. However, ultrasound, while providing less detailed images, has been utilized for measuring the length of the tongue base, which has shown associations with OSA severity. Moreover, endoscopy provides the best solution for obstructive localization.

Considering the varied performances of these imaging techniques, it is imperative to adopt a multifaceted approach for a comprehensive assessment of OSA. Integrating the strengths of each modality, such as the detailed anatomical insights from CT scans and the high-resolution soft tissue visualization from MRI, could enhance the accuracy of OSA detection and characterization. Moreover, combining imaging data with other relevant risk factors, such as BMI and neck circumference, could provide a more holistic understanding of the underlying pathophysiology. Future research endeavor should be directed towards developing non-invasive or low-risk imaging alternatives that can provide precise and comprehensive data for OSA diagnosis, especially for large-scale screening. By addressing these recommendations, the field can advance towards more effective OSA management strategies, facilitating early intervention and improved patient outcomes.

### Negative expiratory pressure

Negative expiratory pressure (NEP) is a technology designed to evaluate airflow limitations and as such its ability to detect UA collapsibility. NEP is obtained by finding the slope of the pressure-flow relationship when applying negative pressure during wakefulness. UA collapsibility is a main anatomical feature of OSA [41]. Based on that, one may predict the severity of sleep-disordered breathing (SDB); thus, different studies have been conducted to study the ability to use NEP features for detecting OSA during wakefulness.

The earliest study for OSA screening using NEP was conducted in 2005 [41], where they investigated the usage of the NEP as a screening tool for SDB, including OSA (moderate and severe) and upper airway resistance syndrome (UARS). Also, a comparison study to find the differences in FVCs of normal expiration and expiration during NEP between various SDBs was done by the same study [41]. Sleep apnea can be differentiated into three types: obstructive. The subjects’ airflow was recorded, while breathing normally and then while breathing with NEP applied during expiration in both sitting and supine positions and with different NEP values. After that, a quantitative index (QI) factor was defined as the ratio of the area under the expiratory FVCs between NEP and normal breathing and was analyzed among the subjects. The study found a significant difference in QI (decreased with the severity of SDB) between the groups after controlling for age, BMI, and expiratory reserve volume via analysis of covariance (ANCOVA). The QI was lower in both values of NEP in most SDB patients than those in healthy controls. The outcomes of this study were congruent with those of medical imaging studies [28, 29] in that FVC features were not found to be as effective as QI in terms of detecting OSA. Moreover, FVCs were considered correlated with OSA if a sawtooth pattern was present [28, 42], though this correlation was greater with overweight subjects than with OSA subjects [28] where the adipose tissue is deposited in the pharyngeal wall [42]. The same outcome has been noted by Sanders et al. [42], where the fluttering of tissue was associated with a sawtooth pattern in the FVC. Another study was performed [43] to study the UA collapsibility of OSA patients using NEP during wakefulness [43]. The same measurement procedure presented in [41] was applied; the results confirmed the outcome of [41] that there was a correlation between the area under the flow-volume curve and UA collapsibility, especially in a seated position.

A similar idea of using NEP for detecting OSA during wakefulness was studied in [44] and [45] in which airway collapsibility was assessed by analyzing expired volume during NEP application. The study in [44] measured the flow drop as a percentage of peak flow immediately after NEP and the expired volume 200 ms after NEP application (V0.2). The results showed that flow drop was significantly higher V0.2 was significantly lower in severe OSA patients, and the predictive effectiveness of flow rate was very high. Moreover, the study in [45] was conducted based on the results of [44] to assess the airway collapsibility by measuring expired volume within different times 200 ms (V0.2), 500 ms (V0.5), and 1 s (V1.0) after NEP. The outcomes were like those in [44] that V0.2 and V0.5 were significantly lower in OSA subjects and that they were both associated with OSA severity.

Another study investigated whether negative pressure (NP)–induced airflow alteration applied to participant’s upper airways during wakefulness was related to OSA severity [46]. While awake, for five full breaths, the 18 participants were orally twice exposed to − 3 cm H2O of NP. Then, the NP ratio (NPR) was calculated as the ratio of the breathing volumes of the last two breaths during NP exposure to the last two breaths before NP exposure. The results showed a strong relationship between participants’ OSA severity (measured by AHI) and the exponentially transformed NPR (ExpNPR) (*R*2 = 0.55, *p* < 0.001). In the multivariable model using the ExpNPR, age, body mass index, and sex as independent variables showed that these variables accounted for 81% of the variability in AHI (*p* < 0.001). Finally, a leave-one-subject-out cross-validation analysis showed that the multivariable model could predict the AHI and it had a strong relation with the actual AHI from PSG (*R*2 = 0.72, *p* < 0.001). Overall, the relationship between ExpNPR and AHI remained robust, independent of demographic factors commonly associated with AHI.

Table 3 presents a summary of all investigated papers in this section. Overall, the application of NEP in evaluating airflow limitations and detecting OSA during wakefulness has emerged as an encouraging technique for predicting the severity of SDB and OSA. NEP allows for the assessment of the pressure-flow relationship during expiration, providing insights into the collapsibility of the upper airway. The discussed studies indicate that NEP features, such as the QI and expired volume measurements, exhibit notable differences between individuals with SDB, including OSA, and control groups even after controlling for various confounding factors. This suggests that NEP holds potential as a reliable and effective method for detecting and assessing UA collapsibility and providing valuable insights into the pathophysiology of OSA during wakefulness.

**Table 3:** Summary of key findings of the investigated papers for NEP

Paper	Sample size	Key result summary
[41]	15 healthy controls, 6 UARS with AHI ≈ 2.5, 9 with 15 ≤ AHI < 30 and 20 with AHI > 30	OSA is characterized by the same or decreased flow during the first 75% of the expiratory period and a lower area under the curve of the NEP flow-volume loop
[43]	20 with AHI < 1.5 and 7 with AHI ≥ 5	A high correlation with the hypotonic and activated slope of pressure-flow relationship measurements. While the seated position showed the strongest correlation
[44]	24 with AHI < 5 and 24 with AHI > 30	OSA is characterized with more flow drop and less volume exhaled. Training area under curve (AUC) of receiver operating characteristics (ROC) was up to 99% with 91.7% sensitivity and 95.8% specificity
[45]	29 with AHI < 5, 28 with 5 ≤ AHI < 15, 34 with 15 ≤ AHI < 30 and 64 with AHI > 30	Exhaled volume percentages at 200 ms and 500 ms were significantly lower in all OSA severity groups. At a threshold of AHI > 15, training AUC of ROC was up to 90% with 93.9% sensitivity and 74.1% specificity
[46]	18 subjects with AHI values between 1.7 and 87.9	The relationship between ExpNPR and AHI remained robust, even when considering demographic factors commonly associated with AHI

Comparatively, while medical imaging techniques offer insights into anatomical changes associated with OSA, NEP appears to focus more directly on the functional aspects of the UA collapsibility, complementing the information provided by imaging technologies. The discussed studies highlight NEP’s ability to capture dynamic changes in airflow during expiration in correlation with the severity of OSA, hence, offering a functional perspective that complements the structural insights provided by imaging techniques. To leverage the full potential of NEP in OSA screening during wakefulness, future research efforts should prioritize the expansion of sample size to enhance the generalizability and robustness of findings.

### Facial images landmarks

**Fig. 6:**
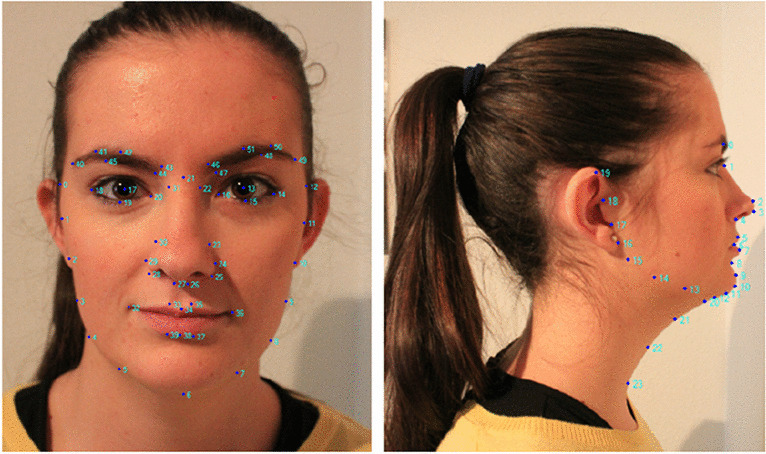
Facial image landmarks, frontal view, and profile [52] (CC BY 4.0)

Recently, with the advancement of digital image processing and computer vision methodologies especially using machine learning and deep learning, researchers have applied these techniques in different medical fields [47] and in particular to OSA detection during wakefulness using facial landmarks  [47, 48]. It is well known that people with craniofacial abnormalities are often diagnosed with OSA [48–50], based on that OSA detection might be possible by using craniofacial characteristics using landmarks from different image postures [51]. Figure 6 shows a standard landmark system used for OSA detection using facial image analysis.

The first research for using facial marks to detect OSA was proposed in [53] and then was enhanced in [54]. In [53], a logistic regression for selections and classification and regression trees were used to predict OSA among 180 subjects (114 as OSA with AHI ≥ 10). The subsequent improvement in [54] was achieved by using the cascade method to automatically detect facial landmarks by using a support vector machine (SVM) classifier to detect the object, and then a cascade regression technique was used for landmark detection [54] in addition to the manual detection used in previous work [53]. The method was applied to 365 subjects (142 controls with AHI < 10 and 223 apneic patients with AHI > 10). The outcomes of the methods showed an accuracy of 70% using manual features (face width, eye width, cervicomental angle, and mandibular length 1) and 69% using automatic landmark detection method. In addition, the authors trained a neural network model for automatic OSA classification with automatic features as input. The neural network model showed an accuracy of 62%.

Another research combined craniofacial morphology points with ML to detect OSA in the Chinese population [55]. However, ethnicity differences as an OSA risk factor may affect facial predictors. The study involved 200 subjects (146 OSA, 54 non-OSA), with calibrated frontal and profile facial photographs collected before PSG. Various facial, demographic, and anthropometrical variables were considered in predicting OSA. Logistic regression modeling showed that cervicomental angle (OR 1.06 per degree; 95% CI, 1.03–1.09; *p* < 0.001) and face width (OR 1.7 per centimeter; 95% CI, 1.1–2.7; *p* = 0.02) were OSA predictors (AUC = 0.76). Tree analysis identified the cricomental space area, mandibular width, mandibular plane angle, and neck soft tissue area as predictors with an AUC of 0.81.

Moreover, recent research employed scanned 3D maxillofacial shapes on 280 Caucasian men with suspected OSA [56]. Alongside PSG, anthropometric data, comorbidities, and medication were also collected at baseline. The valid 3D craniofacial scans of 267 out of 280 subjects were processed using geometric morphometrics and passed to 13 different ML algorithms that were trained and tested. The results showed the ML algorithm achieved a specificity of 56% for detecting those with AHI > 15 (derived from PSG). When combing the 3D geometric data with patients’ anthropometrics, a 0.75 AUC score and sensitivity of 80% with the XGBoost classifier were achieved.

More recently, using pre-trained models from companies such as Vgg from Google [57], different pre-trained facial recognition deep networks have been studied for OSA classification using transfer learning [58]. Since the pre-trained models are trained on the ImageNet dataset and weights and hyperparameters need to be updated, the authors of that study [rf] adjusted the hyperparameters of the pre-trained facial recognition deep networks VGGFace, PAMs-VGG19, and PAMs-AlexNet to classify OSA from facial depth maps (obtained from 3D facial photographs). The results scored a low performance, which was anticipated since the available datasets are very small, while deep learning requires large datasets.

Table 4 presents a summary of all investigated papers in this section. In summary, the integration of digital image processing, computer vision methodologies, and machine learning techniques has ushered in a new era of possibilities in the medical field, particularly in the realm of OSA detection. Notably, the association between craniofacial abnormalities and OSA has motivated the use of facial landmarks as representations of these distinctive craniofacial features, thereby enabling OSA screening during wakefulness. Nevertheless, the accuracy of these technologies has been around 70% [56]. These findings emphasize the need for larger datasets to improve their accuracy.

**Table 4:** Summary of key findings of the investigated papers for facial image landmarks

Paper	Sample size	Key result summary
[53]	66 with AHI < 10 and 114 with AHI ≥ 10	Using clinical variables combined with the photographic measurements resulted in 79.4% validation accuracy, 85.1% specificity, and 69.7% sensitivity
[54]	Dataset 1: 65 with AHI < 10 and 104 with AHI ≥ 10 Dataset 2: 77 with AHI < 10 and 119 with AHI ≥ 10	AUC of ROC of 69% for either automatic or manual marking. A testing classification accuracy of 69.8%, a sensitivity of 68.5%, and a specificity of 76.4%. The logistic regression classifier outperformed the neural network classifier
[55]	200 subjects (54 non-OSA with AHI < 10 and 146 OSA with AHI ≥ 10)	Classification and regression using tree analysis identified cricomental space area, mandibular width, mandibular plane angle, and neck soft tissue area as predictors with an of AUC 0.81
[56]	267 subjects with AHI mean of 23.7 (0.5 to 99.5)	AUC score of 0.75 AUC using the XGBoost classifier when combining 3D geometric data with patient anthropometrics
[58]	69 with an AHI threshold of 15, and 14 for testing	Testing classification accuracy using VGG face, PAMs-VGG19, and PAMs-Alex were 67.42%, 57.14%, and 59.37%, respectively

### Acoustic pharyngometer and nasal airway pressure

**Fig. 7:**
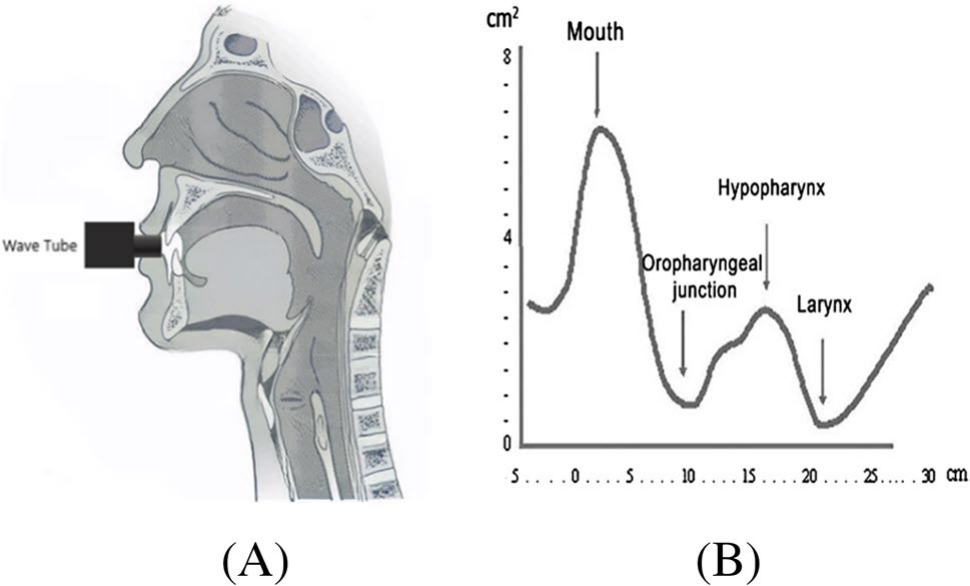
Acoustic pharyngometry: **A** schematic diagram and **B** important anatomical sites [61] (CC BY 4.0)

An acoustic pharyngometer is a non-invasive device that emits acoustic pulses through the mouth into the UA and measures their reflections to determine the minimal cross-sectional area versus distance along the UA [59]. Figure 7 shows the acoustic pharyngometry schematic diagram and recorded signal with related anatomical sites. Since OSA patients have often morphological changes in their UA, it is possible to use pharyngometry during wakefulness [60].

Typically, the pharyngometric measurements are done by performing different UA landmarks (usually 5), while subjects are in upright, supine, and left and right lateral positions [60]. Pharyngometry has been used to measure the minimum cross-sectional area of the UA in 60 OSA subjects in an upright position [62]. The results showed that the minimum cross-sectional area yielded a very good performance in discriminating OSA subjects. Also, by analyzing pharyngometric measurements using different landmarks, [59] the vast majority of measurements in OSA subjects were found to be smaller, while the oropharyngeal junction area measured in the supine position was the most discriminant measurement [59]. These findings agree with the imaging studies [28–30]. The mathematical formulas used in the Kushida Index, as proposed in [60], are derived from a comprehensive analysis of relevant clinical data. These formulas are specifically designed to predict the likelihood of patients developing OSA and to distinguish OSA subjects from controls. The model uses readily obtainable clinical features including measurements of the oral cavity, BMI, and neck circumference. While the exact details of the formulas are beyond the scope of this review, they are based on a study [60] that demonstrated excellent performance in identifying OSA among 300 individuals (46 with AHI < 5 and 254 with AHI > 15). The high sensitivity (97.6%), specificity (100%), positive predictive value (100%), and negative predictive value (88.5%) attest to the reliability of the Kushida Index in identifying OSA risk. In addition, it was demonstrated that the Kushida Index did not correlate with any pharyngometric measurements [59]. The Kushida Index offers a non-invasive and easily implementable approach for screening OSA, particularly in settings where PSG, the gold standard for OSA diagnosis, may not be readily available [59]. Unfortunately, the specific derivation of the formulas was not elaborated. The index’s effectiveness in distinguishing between OSA and non-OSA individuals highlighted its potential utility in clinical practice. However, further research is warranted to validate the index’s performance in diverse populations and to elucidate the underlying mechanisms contributing to its predictive accuracy.

Recently, a study has provided valuable insights into the use of acoustic pharyngometry in preventive otorhinolaryngological programs [63]. The authors compared the anthropometric and pharyngometric measurements of participants aged under and over 40 years in relation to sleep disorders. Then, try to identify the most common oral cavity alterations using acoustic pharyngometry. However, the prevalence of low soft palate and elongated uvula emerged as the predominant oral cavity anomalies pinpointed through acoustic pharyngometry. Additionally, the study revealed notable variations in both anthropometric measures (including BMI, neck circumference, and adjusted neck circumference) and pharyngometric parameters (encompassing cross-sectional area, minimal cross-sectional area, minimal distance, oral cavity length, and volume) between male and female subjects, signifying statistically significant distinctions. Further analysis uncovered compelling positive and negative correlations among these parameters, underscoring the intricate interplay between various physiological factors. Also, the results suggest that acoustic pharyngometry can be a useful tool in the screening and diagnosis of OSA, where the mean cross-sectional areas and airway volumes in any segments are statistically significantly smaller in OSA patients than in healthy controls.

The nasal airway pressure measured in [64] a nasal breathing tube combined with a pressure transducer has also been used to screen OSA during wakefulness [64]; the authors applied nonlinear and nonstationary signal analysis methods on the Hilbert transform of normalized nasal airway pressure signal. The outcome of the research showed a 100% sensitivity and 100% specificity for data set 1 and 85.7% sensitivity and 100% specificity for data set 2 to screen OSA during wakefulness.

Table 5 presents a summary of all investigated papers in this section. In summary, acoustic pharyngometry proves to be a rapid and efficient non-invasive method for assessing the upper airway (UA) in patients with obstructive sleep apnea (OSA). By emitting acoustic pulses and analyzing reflections, the pharyngometer quickly measures minimal cross-sectional area versus distance along the UA. Studies emphasize its discriminant capabilities, particularly in identifying OSA subjects based on the minimal cross-sectional area. Notably, the oropharyngeal junction area, especially in the supine position, emerges as an effective feature for discriminating against OSA subjects. Additionally, detecting nasal airway pressure with a breathing tube and pressure transducer holds promise for efficiently detecting OSA during wakefulness. While these methods show practicality, further investigations with larger cohorts are essential for validation and generalization, ensuring their reliability in clinical OSA assessment and management, ultimately improving patient care and treatment outcomes.

**Table 5:** Summary of key findings of the investigated papers for pharyngometer and nasal airway pressure

Paper	Sample size	Key result summary
[62]	30 with AHI < 15 and 51 with AHI ≥ 15	Cross-sectional area odds ratio 54.21 for multivariate analysis with 87% positive predictive value and 87% negative predictive value
[59]	16 with AHI < 5 and 54 with AHI > 5	Using a Kushida Index of 70 resulted in 89% training sensitivity with 94% specificity
[60]	46 with AHI < 5 and 254 with AHI > 15	The model resulted in 97.6% testing sensitivity with 100% specificity and 99.6% AUC of ROC
[64]	17 with AHI < 5 & 17 with AHI ≥ 5	OSA is characterized by an upward shift in frequencies. The study resulted in 85.7% testing sensitivity with 100% specificity
[63]	100 subjects	Low soft palate and uvula elongation belonged to the most common oral cavity alterations identified by acoustic pharyngometry

### Breathing sound analysis

After successful OSA detection during sleep using only tracheal breathing sounds with or without blood’s oxygen saturation level with very high (~ 96%) accuracy compared to PSG [10, 15, 65], the researchers started to use linear and nonlinear analyses of tracheal breathing sounds recorded in a few minutes during wakefulness to screen OSA [15, 27, 65–74]. The rationale for breathing sound analysis for OSA screening during wakefulness is that structural and morphological changes in the UA, as shown by imaging studies [21], do reflect on the generated breathing sounds in the UA that are detectable by a sensitive microphone and advanced signal processing [70].

One of the first research on this topic investigated the effect of OSA on tracheal sound intensity while breathing normally in both upright and supine positions [75]. The results of sound power analysis showed a higher power of expiratory breathing sounds in the OSA group (AHI > 35) across the frequencies of 200–3000 Hz compared to controls (AHI < 20). This study was one of the first to inspect features of breathing sounds in relation to OSA. However, due to its very small sample size (15 in total), no strong conclusions could be drawn.

Another early study on tracheal breathing sound analysis to screen OSA used formants as the features to classify 10 mild moderate OSA subjects (AHI < 30) from 13 severe OSA subjects (AHI > 30) during wakefulness [76]. The local maxima of the spectral envelope, frequency, relative amplitude, and attenuation of each format, as well as the breath-to-breath variability of these features, were calculated and used as inputs to a linear discriminant analysis (LDA) classifier. The classifier automatically selected the frequency of F4 (say its frequency in Hz here) which was confirmed by the author’s previous studies using six formants analysis [77]. The results show the breath-to-breath variability of its amplitude as its features and obtained a classification accuracy of 77.3% with a sensitivity of 76.9% and specificity of 77.8%. When BMI was added as an additional feature, accuracy increased to 81.8% with a sensitivity of 76.9% and specificity of 88.9% [77]. Another group [65] used a feed-forward neural network to automatically detect breathing segments within a speech recording, and from these breathing segments extracted 104 spectral features like Mel Frequency Cepstral Coefficients (MFCC) to classify 43 OSA subjects (mean AHI 29.1) and 47 controls (mean AHI 4.7). The feature set was reduced to three spectral features using fast-forward selection, and an SVM classifier using these three features achieved 76.5% accuracy with 100% specificity, but only 55% sensitivity which is insufficient for an effective screening tool.

A research group in Manitoba has performed substantial research in breathing sound analysis. In one of their first studies [71], mouth and nose breathing sounds of 35 OSA subjects (AHI > 5) and 17 controls (AHI < 5) were recorded in both the upright and supine positions. Signals were segmented into 50 ms windows, and the variance and median of the power spectrum density (PSD), Katz fractal dimension, and Kurtosis were calculated. Feature selection and reduction were performed using analysis of variance followed by maximum relevancy minimum redundancy (MRMR) algorithm to reduce to two features: (1) median of the average PSD and (2) variance of the average Kurtosis of nasal inspiration in the upright position. LDA and quadratic discriminant analysis classifiers were trained on these two features to discriminate between OSA and non-OSA (AHI threshold of 15), as well as between severe OSA and non-OSA (AHI < 5 or AHI > 30). Quadratic discriminant analysis performed the best in both cases with accuracy, sensitivity, and specificity of 83.3%, 85.0%, 81.3% 91.7%, 92.9%, and 87.5%, respectively. Furthermore, in a subsequent study [27], breathing sound features were compared and combined with anthropometric features for OSA detection. Breathing sounds were recorded from 69 OSA subjects (AHI > 10) and 61 controls (AHI < 5), from which 26 power spectrum-based features were extracted. Unpaired *t*-tests and SVM classifiers were used to reduce the feature set to two features which were then used to train an SVM classifier for OSA classification resulting in 83.9% testing accuracy, 82.6% sensitivity, and 85.2% specificity. Adding anthropometric data such as gender, height, weight, and Mallampati score to the classifier increased these results by only about 1%.

The research was furthered by investigating the relationship between breathing sound features to various anthropometric features [69]. Breathing sounds were recorded in 48 OSA individuals (AHI > 15) and 66 controls (AHI < 15) during wakefulness in the supine position. From this dataset, 412 features were extracted from the PSD, bispectrum, Hurst exponent, and Katz and Higuchi fractal dimensions. A two-step feature reduction phase using *p*-value, area under curve (AUC) of ROC, SVM classification, and correlation coefficients was then performed to reduce the feature set to ten features. For analysis, the subjects were split into two subsets (training and testing) of 105 participants chosen at random (56 non-OSA and 49 OSA) based on sex, BMI, neck circumference, and age for training. Within each subset, each of the 10 features was evaluated using *p*-value, correlation with AHI, and the classification accuracy of SVM classification. Next, a feature selection method based on the coefficients of variation of the AUC of ROC was performed. It was observed that some features had great variability of AUC of ROC and classification accuracy between the subsets. Thus, the conclusion was made that breathing sound features are influenced by anthropometric parameters (sex, BMI, neck circumference, and age) and could, therefore, increase classification performance if anthropometric subsets were selected in the future.

This conclusion was built on in a later study [73], in which two separate feature selection and classification schemes were evaluated in classifying 71 OSA subjects (AHI > 15) and 51 controls (AHI < 15) using 235 extracted breathing sound features. In the first scheme, SVM classifiers were used, and the coefficient of variation and positive impact of the classification accuracies were used to select the features least sensitive to anthropometric variables. SVM classification using various sets of one, two, and three of the least sensitive selected features resulted in maximum classification accuracy of 72.1% (sensitivity 64.7%, specificity 77.5%) using a set of only two features. In the second scheme, subjects were grouped into anthropometric subsets based on age, sex, BMI, and Mallampati score. In this second scheme, the features resulting in the highest classification accuracy in each of the subsets were selected as the most sensitive features to that anthropometric parameter. A new classification method was used in which each subject was classified using four separate SVM classifiers (one for each anthropometric subset), and the results of each classifier were combined using a weighted average. The outcome of the paper claimed a maximum classification accuracy of 83.6%, a sensitivity of 74.5%, and a specificity of 90.1%, by grouping participants according to each anthropometric measure into smaller groups and using a voting process the final result is generated.

Due to the success of the anthropometric-based subset classification, a novel classification algorithm was introduced and tested by the same previous Manitoba research group [70]. Breathing sounds were recorded from 90 OSA subjects (AHI > 15) and 109 controls (AHI < 15) from which 250 features were extracted. Subjects were then split into subgroups as in the previous study, namely training and testing. Feature reduction and selection were done for each subgroup using *p*-values, SVM, and random forest (RF) classifiers to select 3–4 features for each subgroup. For classification, subjects were classified using four RF classifiers (one for each subgroup they fell into), each of which output a decision score: 1 multiplied by the classifier sensitivity for an OSA classification or − 1 multiplied by the classifier specificity for a non-OSA classification. These four decision scores were then summed to obtain the ultimate classification which resulted in 81.4% accuracy with 82.1% sensitivity and 80.9% specificity.

In addition to the SVM and random forest classifiers previously discussed, regularized logistic regression with the least absolute shrinkage and selection operator (LASSO) was also investigated for feature selection and classification [74]. The study used the same subjects as in work [70] but split the subjects into 90 moderate-severe OSA subjects (AHI > 15), 35 mild OSA subjects (5 < AHI < 15) (for testing only), and 74 controls (AHI < 5). A total of 78 PSD-based features were extracted from recorded breathing sounds, as well as 7 anthropometric features which were then reduced to five features via LASSO logistic regression. Performing LASSO linear regression classification on the entire dataset using the five selected features resulted in 81.1% accuracy, 84.4% sensitivity, and 77.0% specificity making it comparable to the previously studied methods. Later LASSO logistic regression and RF methods were compared for both feature selection and classification using the same dataset as in [74]. LASSO logistic regression where it is selected the same feature set as in the previous work [74], while the random forest algorithm selected a similar feature set, but more self-correlated [72]. Based on strictly classification results, RF (accuracy 82.1%, sensitivity 84.2%, specificity 79.5%) outperformed LASSO logistic regression (accuracy 79.3%, sensitivity 82.2%, specificity 75.8%), although logistic regression was faster [72].

Furthering research on the random forest algorithm, the group investigated predicting various additional PSG measurements, aside from only AHI, using breathing sound features [67]. A subset of 145 subjects from previous studies [70] who completed PSG were used in this study, from each of whom 36 PSG parameters were measured. A threshold between OSA and non-OSA was determined for each PSG parameter by comparing the average power spectra of sound signals of subjects above and below various candidate thresholds. The candidate threshold resulting in the greatest gap between the 95% confidence intervals of the average spectra was chosen as the threshold. Unique sound features were selected for each PSG parameter by first removing any features that were highly correlated with other features and then finding the features with the greatest significance between the two groups based on the threshold of the PSG parameter. Then, using a combination of anthropometric and sound features, bilinear polynomial models were developed to estimate each of the PSG parameters resulting in correlation coefficients up to 0.84. The estimated parameters were each individually used as the input to a single feature RF classifier to classify between OSA and non-OSA resulting in classification accuracies up to 88.8% showing definite potential for future use of these predicted parameters for OSA classification [67].

Table 6 presents a summary of all investigated papers in this section. By conceptualizing the UA as an acoustic medium, researchers have aimed to detect physiological abnormalities by analyzing the sound signals generated by breathing during wakefulness. Various studies have explored the potential of different sound features in accurately discerning OSA from non-OSA subjects during wakefulness, providing valuable insights into the diagnostic potential of breathing sound analysis. Initial studies using tracheal breathing sounds during wakefulness started by simply showing the differences in the sounds’ intensity or spectral features between healthy and OSA groups [75, 77]. Later, researchers applied advanced signal processing techniques and classifications to tracheal breathing sounds recorded for a few minutes during wakefulness to identify OSA from non-OSA groups [65, 75, 77]. Overall, the outcomes of these researches highlight the effectiveness of breathing sound analysis as a potential diagnostic tool for OSA, with the successful integration of sound-based parameters pointing towards promising technologies for quick, reliable, and accurate OSA assessment during wakefulness. However, further validation and larger-scale studies are needed to solidify the efficacy and reliability of these methods for potential integration into clinical practice, improving OSA diagnosis and management.

**Table 6:** Summary of key findings of the investigated papers for breathing sounds

Paper	Sample size	Key result summary
[73]	51 with AHI < 15 and 71 with AHI > 15	The least sensitive features to anthropometric factors resulted in 72.1% testing accuracy with 64.7% sensitivity and 77.5% specificity. Most sensitive features to anthropometric factors resulted in 83.6% testing accuracy with 74.5% sensitivity and 90.1% specificity
[69]	66 with AHI < 15 and 48 with AHI > 15	Different risk factors affect the breathing sounds independent of OSA severity. Some sound features showed consistent performance among different anthropometric groups
[70]	109 with AHI < 15 and 90 with AHI > 15	AWakeOSA algorithm resulted in a blind testing accuracy of 81.4%, a sensitivity of 80.9%, and a specificity of 82.1%
[27]	61 with AHI < 5 and 69 with AHI > 10	OSA is characterized by low power at low frequencies (< 350 Hz) and high power at high frequencies (> 350 Hz). Combining anthropometric and sound features resulted in 84.5% validation accuracy with 88.2% sensitivity and 80.9% specificity
[67]	80 with AHI < 15 and 65 with AHI ≥ 15	Many sound and anthropometric features had significant correlations (up to 0.6) with PSG parameters. Developed models had correlations up to 0.84. Testing accuracies to predict PSG parameters were up to 88.8%
[74]	109 with AHI < 15 and 90 with AHI > 15	Testing accuracy of 79.3% with 82.2% sensitivity and 75.8% specificity
[72]	109 with AHI < 15 and 90 with AHI > 15	RF classifier had low variance and outperformed the regularized LR in terms of blind testing accuracy, specificity, and sensitivity with 3.5%, 2.4%, and 3.7% improvement, respectively. The regularized LR was found to be faster than the RF and resulted in a more parsimonious model
[71]	17 with AHI < 5 and 35 with AHI > 5	Individuals with higher AHI had a higher average power spectrum. Using an AHI threshold of 15 resulted in a validation accuracy of 83.3% with 85% sensitivity and 81.25% specificity
[75]	8 with AHI < 20 and 7 with AHI > 35	OSA is characterized by higher inspiratory TSI difference between upright and supine positions
[65]	43 OSA subjects (mean AHI 29.1) and 47 controls (mean AHI 4.7)	Using fast-forward selection and an SVM classifier using these three features obtained 76.5% accuracy, 100% specificity, and 55% sensitivity for testing
[76]	10 with AHI < 30 and 13 with AHI > 30	OSA is characterized by low format frequency in the range of 925 Hz to 1400 Hz. Using LDA resulted in 86.4% validation sensitivity with 88.9% specificity

### Speech sound analysis

Using the same rationale as those studying breathing sounds (that a structural change in the UA should be reflected in the breathing sounds), speech sounds have also been studied for OSA classification. Speaking involves the manipulation of the shape of the UA to produce different sounds, and the way that these manipulations affect the sound and relate to each other has been investigated to detect OSA.

In 2009, early detection of severe apnea cases using effective automatic speech recognition-based detection by employing Gaussian mixture model (GMM)–based speaker recognition technique to distinguish between severe OSA and non-OSA subjects by modeling vowels in nasal and non-nasal phonetic contexts. GMMs were trained using 12 Mel Frequency Cepstral Coefficients (MFCCs) plus energy, as well as their first and second derivatives extracted from voice recordings of four Spanish sentences [78]. Participants in this study consisted of 40 severe OSA (AHI > 30) and 40 controls (AHI < 10); all participants were males, but BMI and age of the OSA group were higher than those of the control group. The results showed that the methodology has an 81% accuracy, 77.5% sensitivity, and 85% specificity. In a follow-up study by the same research group [79], an incremental subset analysis was used to determine the most discriminative features from a set of 16 features extracted from four recorded sentences in Spanish from 62 severe OSA (AHI > 30) and 60 controls (AHI < 10). Both multiple linear regression and LDA were used for classification using the 6, 7, 8, and 9 most discriminative selected features, as well as using all 16 features. The greatest results were obtained using LDA on the eight most discriminative features; which yielded 82.9% accuracy with 85.0% sensitivity and 75.0% specificity. It is worth noting that the test set in this study matched age and BMI between OSA and non-OSA groups, although the training set did not. The authors also studied the correlation between age and BMI with the eight selected speech features and did find a significant correlation among some of the features with both age and BMI but only in the training set. This could indicate that the selected features correlated with AHI also were correlated with age and BMI.

Building further on their previous research, a group investigated the use of both facial image analysis and spectral features of speech to predict subjects’ AHI [51]. MFCCs were extracted from recordings of 258 males with AHI between 0 and 84.4 uttering four Spanish sentences and a set of sustained vowels. The features were transformed using GMMs into a lower dimensional i-vector which was used as the input to a support vector regression (SVR) to estimate AHI. The SVR was tested using various dimensions of i-vectors both with and without clinical variables (age, height, weight, BMI, and cervical perimeter). The best correlation to true AHI using MFCCs and clinical variables was found to be 0.38 with an MAE of 12.43 and was obtained using a 300-dimensional i-vector with clinical variables. This result was no better than when using only clinical variables (correlation of 0.40 and MAE of 12.32) which contrasted the results in other work [78] in which MFCCs were also solely used to predict OSA. This finding thus revealed that MFCCs, alone, may not be a useful tool for OSA screening. AHI estimation using facial features and clinical variables had the best result with a correlation coefficient of 0.45 and an MAE of 11.97, and when using a threshold of AHI = 10, resulted in a classification accuracy of 79.4% with sensitivity of 85.1% and specificity of 69.7%.

This research was furthered by comparing the ability to predict AHI with the ability to predict other clinical variables such as age and BMI from the same speech signals [80]. Nineteen MFCCs and their first derivatives were extracted from recordings of four Spanish sentences and a set of sustained vowels read by 426 male subjects (AHI between 0 and 102). These features were then transformed using GMMs into high-dimensional super vectors and lower-dimensional i-vectors. Next, SVR was used on the super vectors, and various dimensional i-vectors to attempt to predict AHI, as well as age, height, weight, BMI, and cervical perimeter. Overall, the results showed higher correlation coefficients and lower MAE for the estimation of all the anthropometric variables over AHI for different scenarios using super vectors or i-vectors and SVM and SVR. The best AHI prediction was found using a 100-dimensional i-vector with a linear kernel SVR which yielded a correlation of 0.3 and MAE of 13.23.

Two studies investigated the correlation between formant frequencies and their bandwidths with AHI [52, 81]. In one study [81], sustained vowel recordings from 241 males (AHI, 0 to 84) with the same Spanish dialect were used, whereas in a second study [52], sustained vowel recordings from 129 females (AHI, 0 to 108) with the same Spanish dialect were used. In both cases, the Spearman correlation coefficient was used to assess the correlation between the first to third formant frequencies and their bandwidths with AHI. In the male population, only a very weak correlation was found between two of the bandwidths and AHI, but, overall, clinical variables alone provided a stronger correlation, supporting previous findings [51]. For the female population, the frequency of the second formant of /i/ vowel showed a weak correlation with AHI without showing a correlation with any of the clinical variables. Overall, no significant correlations were seen from these studies point towards an underlying correlation between clinical variables and voice features that may provide misleading results in similar studies.

In an earlier study, recordings were taken from 93 subjects uttering short sentences, a set of sustained vowels, and answering yes/no questions in Hebrew [82]. The recordings were manually segmented into 30 ms frames to isolate the vowel sounds and the /n/ and /m/ phonemes. From these segments, 100 short-term and 28 long-term features were extracted, and sequential forward floating selection was used for feature selection among each feature set. Using the selected features with various GMMs, a decision score was calculated for both the short-term and long-term feature sets, and the two scores were then fused using multiplication and 1 nearest neighbor. This yielded a sensitivity of 79% and specificity of 83% for males (using an AHI threshold of 10) and 84% and 86%, respectively, for females (using an AHI threshold of 5). As in studies reviewed [78, 80], the age and BMI of the subjects in this study were both higher among OSA subjects than non-OSA subjects. A later study performed by the same research group of study [82] applied a similar system fusion approach but used different subsystems [83]. In this research, this research used a sustained vowel system, a continuous speech system, and a breathing sound system taken from 208 OSA subjects (AHI > 15) and 190 controls (AHI < 15). The breathing sound system specifically took into account features of the breathing sounds between speaking (i.e., breaths within the speech recordings as in previous work [65]). For the breathing sound system, the forward selection was used to find the most discriminative of a set of 104 extracted features, and then SVR was used to predict AHI. For the sustained vowel system, 12 MFCCs were extracted for each of the vowel sounds, and a convolution neural network (CNN) was used on each set of 12 MFCCs to predict AHI. For the continuous speech system, 12 MFCCs were extracted, and a long short-term memory neural network was used on all 12 MFCCs to estimate AHI. It was found that the best prediction of CNNs for the sustained vowel system results was using vowels /a/ and /u/. Moreover, the prediction of the sustained vowel system was fused with the predictions from the continuous speech and breathing sound systems, and age and BMI to produce a final prediction. Multiple fusion models were tested, and it was found that linear regression with an intersection term provided the best result. AHI was predicted with a Pearson correlation coefficient of 0.61 and an MAE of 8.80. The predicted AHI was also used to classify OSA and non-OSA (using a threshold of AHI = 15), resulting in 77.14% accuracy with 75% sensitivity and 79% specificity.

In another study [84], differentiated severe OSA from healthy subjects using speech signals, one Spanish sentence and a set of sustained vowels from 121 severe OSA subjects (AHI > 30), and 127 healthy controls (AHI < 5) were recorded. Then, 253 features were extracted from the recordings and various techniques for feature reduction and classification. Mann–Whitney *U* test, principal component analysis, LDA, and genetic algorithm (GA) were each used for feature selection, and multi-layer perceptron (MLP), SVM, AdaBoost, *K*-nearest neighbor, and Bayesian classification were each used for classification. Based on fivefold cross-validation, the best-performing feature selection classification pairs were found to be GA with Bayesian classification, GA with SVM, and LDP with MLP. The classifications of these three systems were then combined by a majority vote to produce the final decision yielding 82.9% accuracy, 81.49% sensitivity, and 84.69% specificity in detecting severe OSA. It is worth noting a substantial age difference between groups in the study; the mean age of the severe OSA group was 54, whereas the mean age of the healthy group was 29.7 years. The authors found a Pearson correlation coefficient of 0.60 between age and AHI in the subjects of this study, which could further explain the strong classification results.

More recently, a study focused on studying the use of higher frequency range (> 6 kHz) components of the speech signals and their effect on the detection of OSA during wakefulness [85]. The authors extracted traditional higher-order speech features but added higher-frequency components of the speech signals during awake for a better characterization of OSA patients’ speech. The features included an optimized version of traditional features for higher frequency energy with PCA-based sequence forward feature selection (PCASFFS) for feature selection. The features were extracted from 66 OSA patients. The results show that the new optimized feature for the whole frequency range achieves an accuracy of 84.85% using fivefolds for multi-class OSA detection using the QDA classifier.

Another research studied the nonlinear structure of the OSA subjects’ speech for detection purposes applied to the Turkish population [86]. The characteristics were studied and evaluated for vowels (/a/, /i/, /ı/, and /u/) and 24 consonants (/ca/, /ci/, /cı/, /cu/, /ga/, /gi/, /gı/, /gu/, …etc.); then, different trials were applied to search in which voice group the nonlinear features were more discriminant in OSA. The nonlinear analysis was applied to a wide variety of voice samples having vocal tract components recorded from 40 subjects (20 OSA and 20 healthy subjects). The results showed the consonants to be more effective for classification than the vowels. Using the whole dataset and employing fivefold cross-validation, the best OSA detection performance using vowels was 83.5% using KNN, and the best performance using consonants only was 96% using SVM. Moreover, 82.5% accuracy was achieved with only six features from consonants using KNN on a blind subset of data. The study supports the hypothesis that the nonlinear characteristics of vocal tract changes in subjects with OSA.

Table 7 presents a summary of all investigated papers in this section. Many studies have explored the use of speech sound analysis for classifying and predicting OSA, leveraging changes in the upper airway structure due to OSA that can affect speech sounds. Various features extracted from speech recordings demonstrate the potential of speech sound analysis to effectively discern OSA patients using machine learning techniques and feature selection methods [79–81]. Overall, comprehensive speech sound analysis shows promising potential for effective OSA detection and prediction, with multimodal approaches and advanced machine learning techniques providing robust results. Further research and larger-scale studies are necessary to solidify the reliability and clinical applicability of these methods.

**Table 7:** Summary of key findings of the investigated papers for speech analysis

Paper	Sample size	Key result summary
[81]	241 males with AHI between 0 and 84	Weak correlations (up to 0.21) were seen between AHI and voice features (formants)
[79]	60 with AHI < 10 and 62 with AHI > 30	The eight most discriminative features with LDA result in 82.9% accuracy with 85.0% sensitivity and 75.0% specificity for testing. Also, it is noted that the test set in this study matched age and BMI between OSA and non-OSA groups, while the training set did not
[80]	426 male subjects with AHI between 0 and 102	19 MFCCs and their first derivatives were transformed using GMMs into high-dimensional super vectors and lower-dimensional i-vectors. The best AHI prediction was found using a 100-dimensional i-vector with a linear kernel SVR which yielded a maximum correlation of 0.3, and the testing classification accuracy was 71% with 92% sensitivity and 20% specificity
[51]	258 males with AHI between 0 and 84.4	Transformed MFCCs using GMMs into high-dimensional super vectors and lower-dimensional i-vectors used as the input to an SVR, the best correlation was found to be 0.45 with a testing classification accuracy of 79.4% with 85.1% sensitivity and 69.7% specificity
[78]	40 with AHI < 10 and 40 with AHI > 30	12 Mel Frequency Cepstral Coefficients (MFCCs) plus energy, as well as their first and second derivatives extracted and then passed to GMMs results in 81% accuracy, 77.5% sensitivity, and 85% specificity for validation
[82]	Males (12 with AHI < 10 and 48 with AHI > 10) Females (14 with AHI < 5 and 19 with AHI > 5)	From 30 ms segments, 100 short-term and 28 long-term features were extracted, and sequential forward floating selection was used for feature selection among each feature set. The method yielded a testing sensitivity of 79% and testing specificity of 83% for males, and 84% and 86%, respectively for females
[83]	190 with AHI < 15 and 208 with AHI > 15	12 MFCCs were extracted for each of the vowel sounds, and a CNN to predict AHI. Then, the predicted AHI was also used to classify OSA and non-OSA (using a threshold of AHI = 15), resulting in a correlation of 0.61 between AHI and the fused predicted AHI and 77.1% accuracy with 75% sensitivity and 79% specificity for testing
[84]	127 with AHI < 5 and 121 with AHI > 30	253 features from the recordings and tested various techniques for feature selection and classification. The best feature selection classification pairs were found to be GA with Bayesian classification, GA with SVM, and LDP with MLP. Then by combining these by a majority vote yielding 82.9% accuracy, 81.49% sensitivity, and 84.69% specificity in detecting severe OSA for validation
[52]	129 females (AHI 0 to 108.4)	Formant frequencies showed a weak correlation (up to − 0.26) with AHI
[85]	66 Subjects (31 subjects for AHI < 5; 13 subjects for 5 ≤ AHI < 15;10 subjects for 15 ≤ AHI < 30; and 12 subjects for AHI ≥ 30)	The new optimized feature for the whole frequency range achieves an accuracy of 84.85% for multi-class OSA detection using the QDA classifier
[86]	40 subjects (20 healthy and 20 OSA with AHI > 9)	The nonlinear characteristics of vocal tract changes in subjects with OSA can be used as discriminant features, especially for consonants. Vowels feature only were 83.5% using KNN; consonants only were 96% using SVM; and 85.5% accuracy using a blind test set with six consonant features

### Questionnaires

Many studies have been published to evaluate the performance of OSA screening during wakefulness by questionnaires [87]. Here, we review the studies on the reliability of OSA screening using the following questionnaires: the Epworth Sleepiness Scale (ESS), the Berlin questionnaire, the STOP-Bang questionnaire, and the STOP questionnaire [18, 88]. A meta-analysis was applied to evaluate and compare the clinical screening tests of OSA and build a case for using them before surgery. For each screening test in this evaluation, diagnostic odds ratios were utilized as summary metrics of accuracy, and false-negative rates were used as markers of missed diagnosis [87]. The results reveal that test accuracy in many validation studies of the same screening test is inconsistent, suggesting an underlying heterogeneity in either the clinical presentation or the measured clinical components of these models. Moreover, the false-negative rates show that a large fraction of patients with OSA were missed by most clinical screening tests [87].

For screening the OSA using questionnaires, a study was conducted [89], the study assessed the questionnaire’s capability to detect increased apnea activity, and an epidemiologic investigation of OSA with 465 people was conducted. A questionnaire consisting of 56 questions about sleeping patterns, feeling sleepy, and performance during the day and an in-home sleep study was completed by subjects and their roommates separately. The responses were analyzed using LR, factor analysis, and ROC. The results of factor analysis showed that 16 questions, grouped into five variables (functional impact of drowsiness, self-reported breathing abnormalities, roommate-observed breathing disturbances, driving impairment, and insomnia), were found to account for 67% of the variance in the questionnaire data. Moreover, for nine out of ten questions, there was some degree of agreement between the subject’s and his or her partner’s self-reported responses (kappa statistics, 0.34 to 0.57). Also, three questions about snoring intensity, choking a roommate saw, and dozing off while operating a vehicle were found to be the most accurate predictors of increased apnea activity, according to logistic regression analysis (ROC area, 0.78). Symptoms combined with information on gender and BMI increased prediction power by 10% (ROC area, 0.87). In population surveys of OSA, questionnaire data thus offer a reliable method of characterizing symptom distributions. Multiple questions or a separate roommate questionnaire do not greatly improve predictive ability, but gender and BMI information do.

Another study to evaluate the performance of the STOP-Bang questionnaire as a screening tool for OSA was conducted [19]. They evaluate the studies that have been done on adults, the performance of the results was validated using PSG, and the subject’s OSA was defined as AHI ≥ 5. A systematic review of 17 studies with a total number of 9206 patients was done; the results showed that the STOP-Bang questionnaire has a sensitivity value of 90% for any OSA (AHI ≥ 5), while the sensitivity increased for higher AHI values to 94% for moderate-to-severe OSA (AHI ≥ 15) and 96% severe OSA (AHI ≥ 30). Moreover, the NPV was 46%, 75%, and 90% for the same AHI values, respectively. While, in the sleep clinic population, 25% of the severe OSA have a STOP-Bang score of 3 with rising proportionally probability of 35%, 45%, 55%, and 75% with a stepwise increase of score to 4, 5, 6, and 7/8. The analysis concluded by demonstrating that the likelihood of having moderate-to-severe OSA increases with increasing the STOP-Bang score.

A study to evaluate the STOP-Bang questionnaire was done [88]; the authors used a dataset of 856 subjects attending a sleep clinic for PSG which was used to evaluate the performance of the questionnaire. The authors used four of eight STOP-BANG questionnaire features, and these features were combined using a logistic regression (LR) model, where the model performance was evaluated using 80–20% train and test sets. The outcome of the study in the test set was 83.3%, 45.8%, 62.2%, and 71.7% for sensitivity, specificity, positive predictive value, and negative predictive value, respectively, while the AUC has a mean value of 0.717. Moreover, it is also shown that a subset of STOP-Bang questionnaire features has the same performance as the full features set which means that there are redundant questions/features in the STOP-Bang questionnaire. A cross-sectional study to compare different questionnaires for screening of OSA was conducted [18], the study included 234 patients attending the sleep clinic for overnight PSG, and then the patients were administered four sleep questionnaires (Berlin, Epworth Sleepiness Scale [ESS], STOP, and STOP-Bang). The results showed that the STOP-Bang has the highest sensitivity among all OSA severity categories (97.55%, 97.74%, and 98.65% for OSA, moderate-to-severe, and severe, respectively), then the Berlin (95.07%, 95.48%, and 97.3%) and STOP questionnaires (91.67%, 94.35%, 95.48%, and 95.95%). While regarding the specificity, ESS had the highest value to predict OSA (75%), moderate-to-severe OSA (48.15%), and severe OSA (46.43%) but with the lowest sensitivity values. Finally, the Berlin, STOP, and STOP-Bang questionnaire sensitivity was quite high, but because of their low specificity, they produced more false-positive results and failed to exclude those who were at low risk. Moreover, to determine which patients are at risk and to determine the ideal combination of these tools, the clinical utility of five different questionnaires—STOP, STOP-Bang, Berlin questionnaire, Epworth Sleepiness Scale, and 4-Variable Screening Tool—in a sleep clinic is being evaluated [90]. Like in the previous studies, the outcome shows that the highest specificity was found in 4-V, while SB had the highest sensitivity and AUC. Their predictive value was not increased by combining various surveys. Yet, another more recent study compared the reliability of different questionnaires in the detection of OSA on 201 subjects with different OSA severity, where the subjects completed five different types of questionnaires: the ESS questionnaire, the STOP-Bang questionnaire, the STOP questionnaire, the BQ questionnaire and the Pittsburgh Sleep Quality Index (PSQI) [91]. Moreover, the subjects were examined using limited PSG, and the performance of the questionnaires was evaluated. The results showed the highest sensitivity was achieved by STOP-Bang (81.6%), Berlin (78.7%), and STOP questionnaires (74.2%), while the PSQI and ESS’ sensitives were low (50.8% and 34.5%, respectively). For specificity, the highest values were achieved by ESS (82.6%), STOP-Bang (75%), STOP (61.9%), and Berlin questionnaires (61.9%). Based on the results, the STOP-Bang and Berlin questionnaires were found to be the most reliable screening tool. Also, the STOP questionnaire was found to have the most time-saving nature as is a short questionnaire.

Table 8 presents a summary of all investigated papers in this section. Many studies have evaluated the performance of common OSA screening questionnaires like the ESS, Berlin questionnaire, STOP-Bang, and STOP questionnaire, revealing variations in accuracy and false-negative rates. While some studies highlighted specific predictors within questionnaires, others emphasized potential redundancies. Comparative evaluations consistently showed high sensitivity but very low specificity, leading to increased false positives and the inability to exclude low-risk individuals. Recent research identified STOP-Bang and Berlin questionnaires as having the highest sensitivity, while ESS exhibited the highest specificity. Despite their sensitivity, ongoing research and refinement are essential to address specificity limitations and optimize the clinical utility of OSA screening tools.

**Table 8:** Summary of key findings of the investigated papers for the questionnaire technique

Paper	Sample size	Key result summary
[87]	26 different studies	Testing results: for AHI of 5, sensitivity (28.6%, 83.6%) and specificity (38.2%, 68.5%); for AHI of 10, sensitivity (30.4%, 77.8%) and specificity (64.2%, 98.8%); for AHI of 25, sensitivity (85.1%, 88.2%) and specificity (75.9%, 81.2%); and for AHI of 30, sensitivity (61.4%, 100%) and specificity (36%, 75%)
[88]	155 with AHI < 5, 106 with 5 ≥ AHI < 15, 123 with 15 ≥ AHI < 30, 215 with AHI ≥ 30, and 257 with snore	A performance of 83.3%, 45.8%, 62.2%, 71.7%, and 0.717 for sensitivity, specificity, positive predictive value, negative predictive value, and AUC, respectively
[18]	234 with AHI < 5, 27 with 5 ≥ AHI < 15, 177 with 15 ≥ AHI < 30, and 148 with AHI ≥ 30	Testing results: for AHI of 5, sensitivity (91.7%, 97.6%) and specificity (25%, 26.3%); for AHI of 15, sensitivity (75.7%, 97.7%) and specificity (3.7%, 48.2%); for AHI of 30, sensitivity (79.7%, 98.7%) and specificity (5.4%, 46.4%)
[89]	184 control subjects and 281 OSA subjects	16 questions were found to account for 67% of the variance in the questionnaire data. Moreover, for nine out of ten questions, a degree of agreement between the subject's and his or her partner’s self-reported responses (kappa statistics, 0.34 to 0.57). Finally, combined with gender and BMI increased prediction by 10% (ROC area, 0.87)
[19]	17 articles with a 9206 total number of subjects	Using STOP-Bang threshold of 3 for the clinic and surgical populations, listed respectively in the following: For AHI ≥ 5, 90 and 84% sensitivity and 49 and 43% specificity; for AHI ≥ 15, 94 and 91% sensitivity and 34 and 32% specificity; for AHI ≥ 30 were 96 and 96% sensitivity and 25 and 29% specificity
[90]	1853 patients	Testing results: for AHI of 5, sensitivity (50%, 95%) and specificity (14%, 78%); for AHI of 15, sensitivity (54%, 97.6%) and specificity (12.7%, 74.4%); for AHI of 30, sensitivity (57%, 98.7%) and specificity (9.9%, 69.3%)
[91]	24 with AHI < 5, 28 with 5 ≤ AHI < 15, 65 with 15 ≤ AHI < 30, and 84 with AHI ≥ 30	STOP-Bang and Berlin’s questionnaires demonstrated the highest sensitivity (81.6% and 78.7%, respectively), while ESS exhibited the highest specificity (82.6%)

## Challenges

This section investigates the challenges in OSA detection methods used during wakefulness.

### Sample size

The sample size is an important part of any study; for OSA detection, this is one of the major issues since it is hard to collect other data from patients especially before and after the PSG recording [92]. Even though, recording PSG is hard to record since it is costly and requires the patient to sleep at the hospital sleep lab [14]. Most of the studies are performed on a relatively small sample size, and in many cases, the recorded datasets are imbalanced which causes biasing on the results towards one of the classes. Based on that, any future studies must include larger datasets.

### Affordability

The cost of the process to diagnose patients with OSA is high when it is referred to PSG, imaging studies, and NEP [29, 37, 43]. Another process like detection using breathing sounds and speech sounds is very cheap [73]. Moreover, researchers in their future research methods should ensure that their proposed systems are affordable. The effective parameters on the affordability consist of the technology of the process, fabrication materials, required equipment, and the transmission technology if required.

### Ease of use

Usually, OSA diagnosis devices are not easy to use and require a complex setup of the process and a specialized person for the interpretation of the acquired data [43]. This is a major challenge to the current methodologies; based on that, researchers must take into consideration the simplicity of the process setup and in it is best cases the patient can set it up on their side with easy instructions.

### Portability

The portability of any proposed system for wakefulness detection is OSA which is a main challenge, since most of the reviewed systems and methods required high computational processes when using data processing [93]. Moreover, some techniques like medical imaging require large equipment for data acquisition [28]. Based on that, researchers in the future must be able to use cloud computing and wireless transmission of data to overcome such challenges.

### Measurement time

Measuring time is one of the issues during studies and affects the number of the included subjects in the studies [40]. Moreover, it also affects the ability to apply any system in real time [53]. Also, since our main focus in this review is on the detection of OSA during wakefulness, it is important and challenging to make proposed methods to record required measurements in a short time and even generate the results in a short time [93]. Any future research should focus on the required time for measuring as one of the main challenges to be overcome.

### Detection of different OSA severity

Most of the proposed methods are focused on detecting if there is OSA or not based on a threshold applied to the recorded AHI [94]. One of the challenging things in the future development of OSA detection during wakefulness is to provide a system that can detect the severity of the OSA based on the predefined AHI value thresholds [45]. Such a system can help provide a full diagnosis system instead of a classification system.

### Performance

Developing an advanced and high-performance method that is able to detect OSA with very high performance is the ultimate goal of any detection and classification system [42]. Additionally, based on the previously discussed challenges, this can be quite challenging based on the number of subjects included in the studies [82]. So, the performance of the detection system also remains an important challenge by focusing on the specificity and sensitivity not only the accuracy and needs to be investigated further in-depth in any future work.

### Providing physiological interpretation and information beyond AHI

The primary outcome of the PSG system is the apnea/hypopnea index (AHI) which is the primary to detect OSA severity. However, other severity parameters like total arousal index and SpO2 are very important to provide a full diagnosis of the patient and decide on a treatment option [67]. PSG assessments and home sleep tests measure these parameters, but most wakefulness techniques are unable to estimate or predict these parameters; there has been only one study [67]. In future methods, there is a need to provide a system able to estimate or predict these parameters and to be investigated further in-depth in any future work.

## Managing false positives in OSA detection

False positives can emerge in various OSA detection techniques; for each of these techniques, there are different reasons behind false positives, and different guidance on managing an excessive number of clinically irrelevant OSA detections is needed. These insights are essential for researchers, clinicians, and technologists striving to enhance the accuracy and reliability of OSA diagnosis during wakefulness [95]. By addressing the issue of false positives systematically across different OSA detection techniques, we aim to contribute to the development of more precise and clinically relevant methods [96]. The goal is to ensure that patients receive accurate diagnoses, appropriate treatment plans, and peace of mind, while healthcare resources are utilized efficiently and effectively. In the following, we will delve into specific techniques and their respective strategies for managing false positives [97].

For the use of imaging techniques, mitigating clinically irrelevant OSA detections involves implementing robust post-processing methods and automatically identifying and excluding artifacts [96]. It is crucial to set specific parameters during image acquisition and establish criteria for extracting anatomical features based on validated clinical data to distinguish between relevant and irrelevant findings. Regular calibration of imaging equipment, adherence to standardized protocols, and employing standard device setups are essential to minimize false positives [95, 97].

For various OSA detection methods, managing excessive clinically irrelevant detections necessitates specific strategies. In NEP tests, clear clinical guidelines defining thresholds for collapsibility and guiding repeat tests, or different interpretations are crucial [95]. Training healthcare professionals in NEP interpretation nuances can further reduce the likelihood of excessive irrelevant detections [97]. In facial landmarks analysis, refining algorithms and incorporating machine learning models based on large datasets enhance landmark detection accuracy [98]. Similar precision improvements can be achieved in pharyngometry by establishing normative data for airway dimensions, considering dynamic changes during sleep, and comparing patient data to norms [95, 97].

Advanced signal processing in breathing sound analysis, including using balanced groups dataset, appropriate recording protocols, and patient-specific characteristics, enhances accuracy [96, 99, 100]. Similarly, in speech signal analysis, focusing on specific speech features, considering contextual information, and employing continuous monitoring and real-time feedback systems contribute to accuracy [98, 99]. In questionnaires, refining designs, implementing scoring thresholds, and combining questionnaire data with physiological parameters improve diagnostic accuracy and reduce irrelevant detections [96, 99, 100]. Overall, integrating these tailored strategies into each OSA detection technique enhances precision, reliability, and clinical relevance [96].

## Discussion

Gold standard OSA diagnosis, an overnight PSG sleep study, has many drawbacks such as being labor-intensive, time-consuming, expensive, and lack of availability in remote areas. Thus, research interest in detecting OSA during wakefulness within a few minutes has been on the rise, especially in the last decade. This review has been dedicated to reviewing the studies dedicated to understanding OSA manifestation on the upper airway as well as technologies to screen and detect OSA during wakefulness. This review has presented 57 journal papers and conference papers; all papers related to screening children were excluded since children, and adults have significant disparities in sleep and respiratory physiology and their OSA pathology [101].

Having analyzed and condensed available literature, characteristics of a good OSA screening tool have been identified as (1) affordability, (2) ease of use, (3) portability, (4) executability during wakefulness, (5) prompt setup and measurement time, (6) large sample size testing, (7) non-invasiveness, (8) ability to screen for different OSA severity groups, (9) accuracy with high sensitivity and specificity, and (10) ability to provide physiological interpretation and information beyond AHI. Most of these characteristics are a challenge that faces the past and current development of OSA wakefulness technologies. Given these characteristics, imaging techniques would not meet the design specifications for a future OSA screening tool, as imaging methods remain bulky, expensive, and not readily available outside of a clinical setting. However, imaging techniques remain very helpful research tools to better understand the pathogenes of the disorder. Table 9 summarizes the investigated papers’ method characteristics. Of the 57 reviewed papers, 40 papers proposed a classification analysis methodology, while only 12 papers of these 40 introduced only training results, 11 papers introduced validation results, and 25 papers introduced testing results. Moreover, the number of participants per study was between 14 [36] and 597 [80] individuals, and this number is still small given the heterogeneity of the OSA population and its confounding variables and also compared to the number of samples that application of artificial intelligence and deep learning required to achieve reliable results.

**Table 9:** Summary of methodology in reviewed works based on method characteristics

Reference	Author	Year	Afford	Port	Wake	Prompt	Sample	Tested	Non-inv	Groups	Spec	Sens	More
			≤ US$5000	Easily transported to home or clinic	Used during wakefulness	≤ 15 min measurement time	*N* ≥ 100 and *N* of one severity group is ≥ 30	Evaluated a blind testing dataset	Non-invasive technology	Can screen for ≥ 2 OSA severity groups	≥ 80%	≥ 80%	Additional metrics can be measured
[42]	Sanders MH, Martin RJ, Pennock BE, Rogers RM	1981	Y	Y	Y	Y	N	N	Y	N	Y	Y	N
[29]	Haponik EF, Smith PL, Bohlman ME, Allen RP, Goldman SM, et al	1983	N	N	Y	N	N	N	Y	N	N	N	Y
[28]	Riley R, Guilleminault C, Herran J, Powell N	1983	N	N	Y	Y	N	N	Y	N	N	N	Y
[30]	Schwab RJ, Gefter WB, Hoffman E a, Gupta KB, Pack AI	1993	N	N	Y	N	N	N	Y	N	N	N	Y
[75]	Pasterkamp H	1996	Y	Y	Y	Y	N	N	Y	N	N	N	N
[60]	Kushida CA, Efron B, Guilleminault C	1997	Y	Y	Y	Y	Y	Y	Y	N	Y	Y	N
[37]	Schwab RJ, Pasirstein M, Pierson R, Mackley A, Hachadoorian R, et al	2003	N	N	Y	N	N	N	Y	N	N	N	Y
[59]	Dae GJ, Hae YC, Grunstein RR, Yee B	2004	Y	Y	Y	Y	N	N	Y	N	N	N	Y
[41]	Tamisier R, Wuyam B, Nicolle I, Pépin JL, Orliaguet O, Perrin CP	2004	Y	Y	Y	Y	N	N	Y	N	N	N	N
[64]	Salisbury JI, Sun Y	2006	Y	Y	Y	Y	N	Y	Y	N	Y	Y	N
[40]	Lahav Y, Rosenzweig E, Heyman Z, Doljansky J, Green A, et al	2009	Y	Y	Y	Y	N	N	Y	N	N	N	Y
[53]	Lee RWW, Petocz P, Prvan T, Chan ASL, Grunstein RR, Cistulli PA	2009	Y	Y	Y	Y	Y	N	Y	N	Y	N	N
[78]	Pozo RF, Murillo JLB, Gmez LH, Gonzalo EL, Ramírez JA, et al	2009	Y	Y	Y	Y	N	N	Y	N	Y	N	N
[87]	Ramachandran SK, Josephs LA	2009	Y	Y	Y	Y	-	Y	Y	Y	N	Y	N
[93]	Caseiro P, Fonseca-Pinto R, Andrade A	2010	Y	Y	Y	Y	N	N	Y	N	N	N	N
[44]	Romano S, Salvaggio A, Hirata RP, Bue A Lo, Picciolo S, et al	2011	Y	Y	Y	N	N	N	Y	N	N	N	N
[45]	Romano S, Salvaggio A, Bue A Lo, Marrone O, Insalaco G	2011	Y	Y	Y	N	N	N	Y	Y	N	N	N
[82]	Goldshtein E, Tarasiuk A, Zigel Y	2011	Y	Y	Y	Y	N	Y	Y	N	Y	Y	N
[71]	Montazeri A, Giannouli E, Moussavi Z	2012	Y	Y	Y	Y	N	N	Y	N	Y	Y	N
[18]	El-Sayed IH	2012	Y	Y	Y	Y	-	Y	Y	Y	N	Y	N
[62]	DeYoung PN, Bakker JP, Sands SA, Batool-Anwar S, Connolly JG, Butler JP	2013	Y	Y	Y	Y	N	N	N	N	Y	Y	Y
[76]	Solà-Soler J, Fiz JA, Torres A, Jané R	2014	Y	Y	Y	Y	N	N	Y	N	Y	Y	N
[84]	Solé-Casals J, Munteanu C, Martín OC, Barbé F, Queipo C, Amilibia J	2014	Y	Y	Y	Y	Y	N	Y	N	Y	Y	N
[43]	Carrera HL, Marcus CL, McDonough JM, Morera JCO, Huang J, Farre R	2015	Y	Y	Y	N	N	N	Y	N	N	N	N
[79]	Montero Benavides A, Fernández Pozo R, Toledano DT, et al	2014	Y	Y	Y	Y	Y	Y	Y	N	N	Y	N
[90]	Pataka A, Daskalopoulou E, Kalamaras G, Fekete Passa K, et al	2014	Y	Y	Y	Y	-	Y	Y	Y	N	Y	N
[51]	Espinoza-Cuadros F, Fernández-Pozo R, Toledano DT, Alcázar-Ramírez JD, López-Gonzalo E, Hernández-Gómez LA	2015	Y	Y	Y	Y	Y	Y	Y	N	N	Y	N
[19]	Nagappa M, Liao P, Wong J, Auckley D, Ramachandran SK, et al	2015	Y	Y	Y	Y	-	Y	Y	Y	N	Y	N
[80]	Espinoza-Cuadros F, Fernández-Pozo R, Toledano DT, et al	2016	Y	Y	Y	Y	Y	Y	Y	N	N	Y	N
[81]	Montero Benavides A, Blanco Murillo JL, Fernández Pozo R, et al	2016	Y	Y	Y	Y	Y	N	Y	N	N	N	N
[88]	Behar JA, Palmius N, Daly J, Li Q, Rizzatti FG, et al	2017	Y	Y	Y	Y	-	Y	Y	Y	N	Y	N
[54]	Balaei AT, Sutherland K, Cistulli PA, De Chazal P	2017	Y	Y	Y	Y	Y	Y	Y	N	N	N	N
[27]	Elwali A, Moussavi Z	2016	Y	Y	Y	Y	Y	N	Y	N	Y	Y	Y
[52]	Tyan M, Espinoza-Cuadros F, Pozo RF, Toledano D, Gonzalo EL, Ramirez JDA	2017	Y	Y	Y	Y	Y	N	Y	N	N	N	N
[89]	Kump K, Whalen C, Tishler P V., Browner I, Ferrette V, et al	1994	Y	Y	Y	Y	-	Y	Y	Y	N	Y	N
[36]	Darquenne C, Elliott AR, Sibille B, Smales ET, DeYoung PN, et al	2018	N	N	Y	N	N	N	Y	N	N	N	Y
[58]	Islam SM, Mahmood H, Al-Jumaily AA, Claxton S	2018	Y	Y	Y	Y	N	Y	Y	N	N	N	N
[65]	Simply RM, Dafna E, Zigel Y	2018	Y	Y	Y	Y	N	Y	Y	N	Y	N	N
[69]	Elwali A, Moussavi Z	2019	Y	Y	Y	Y	Y	N	Y	N	N	N	N
[74]	Hajipour F, Jozani MJ, Elwali A, Moussavi Z	2019	Y	Y	Y	Y	Y	Y	Y	N	N	Y	Y
[73]	Elwali A, Meza-Vargas S, Moussavi Z	2019	Y	Y	Y	Y	Y	Y	Y	N	N	Y	N
[70]	Elwali A, Moussavi Z	2017	Y	Y	Y	Y	Y	Y	Y	N	Y	Y	Y
[83]	Simply RM, Dafna E, Zigel Y	2020	Y	Y	Y	Y	Y	Y	Y	N	N	N	N
[72]	Hajipour F, Jozani MJ, Moussavi Z	2020	Y	Y	Y	Y	Y	Y	Y	N	N	Y	N
[67]	Elwali A, Moussavi Z	2021	Y	Y	Y	Y	Y	Y	Y	N	Y	Y	Y
[32]	Molnár V, Molnár A, Lakner Z, Tárnoki DL, Tárnoki ÁD, Jokkel Z, et al	2023	N	N	Y	N	N	N	Y	N	Y	Y	Y
[33]	Bindi I, Ori M, Marchegiani M, Morreale M, Gallucci L, Ricci G	2022	N	N	Y	N	N	N	Y	N	N	N	N
[35]	Molnár V, Lakner Z, Molnár A, Tárnoki DL, Tárnoki ÁD, Kunos L, et al	2022	N	N	Y	N	N	N	N	Y	Y	Y	Y
[34]	Molnár V, Lakner Z, Molnár A, Tárnoki DL, Tárnoki ÁD, Kunos L, et al	2022	N	N	Y	N	N	N	Y	N	Y	Y	Y
[46]	Lim J, Alshaer H, Ghahjaverestan NM, Bradley TD	2023	Y	Y	Y	N	N	N	Y	Y	N	N	N
[55]	Sutherland K, Lee RWW, Petocz P, Chan TO, Ng S, Hui DS, et al	2016	Y	Y	Y	Y	Y	Y	Y	N	N	N	N
[56]	Monna F, Ben Messaoud R, Navarro N, Baillieul S, Sanchez L, Loiodice C, et al	2022	Y	Y	Y	Y	Y	Y	Y	N	N	N	N
[63]	Shivarov G	2022	Y	Y	Y	Y	N	N	Y	N	N	N	N
[85]	Pang K-G, Hsung T-C, Liao G, Ling W-K, Law AK-W, Choi WS	2022	Y	Y	Y	Y	Y	-	Y	-	-	-	-
[86]	Yılmaz D, Yıldız M, Uyar Toprak Y, Yetkin S	2023	Y	Y	Y	Y	Y	Y	Y	N	Y	Y	Y
[91]	Solecka Š, Matler K, Kostlivý T, Kubec V, Tomášková H, Betka J	2022	Y	Y	Y	Y	Y	Y	Y	Y	Y	Y	Y

A major drawback with imaging techniques [28–30, 36, 37, 58], negative expiratory pressure [41, 43–45], and pyranometer-based studies [59, 60, 62] is that they did not introduce any testing classification results; these studies require further investigation with validation and blind testing results. On the other hand, facial-related papers provided testing classification accuracies, but they were relatively low: they were between 57.14% [58] using deep learning and 69.8% [54] using automatics landmark detection with NN classification. These results show that facial imaging still needs more development and may require combining extracted features from these techniques with other features such as anthropometric features to enhance the overall performance. In contrast to the above studies, the OSA detection performance was increased in breathing sound–related papers, with testing classification accuracies between 72.1 and 83.6% [73], sensitivities between 55 [65] and 82.2% [72], and specificities between 75.8 [72] and 100% [65]. While tracheal breathing sound analysis has shown reasonably high blind test sensitivity and specificity, studies have shown the accuracy can still be benefited by combining some anthropometric features with the sound analysis [70]. Similar to breathing sounds, speech sound analysis can also be used for OSA detection. More variation was noticed in speech signal–related papers, with testing classification accuracies between 71 [80] and 79.4% [78], sensitivities between 75 [79] and 92.9% [71], and specificities between 20 [80] and 79% [83]. On the other hand, the greatest variation was seen in papers related to questionnaires, showing testing sensitivities between 30.4 [19] and 99.8% [18] and specificities between 3.7 [18] and 98.8%. The oral cavity and clinical measurements related article provided 97.6% testing sensitivity and 100% specificity [60]. However, the oral cavity and clinical measurement model has certain limitations that affect its accuracy and further development. These limitations arise from extreme values in the model’s variables and include factors such as age restrictions (persons younger than 15 years or older than 80 years), conditions like Marfan syndrome or major muscle disorders, oral abnormalities (cleft palate, severe malocclusion, or reconstructive surgery), coexisting serious medical conditions, and limited ethnic diversity in the patient sample. Air pressure–related papers provided 85.7% testing sensitivity and 100% specificity [64]. However, the air pressure research paper was done on very small datasets; thus, further investigation and standardizing the instrumentation are required to confirm the robustness of the proposed methodology. Furthermore, there is interest in predicting other OSA-related parameters that a PSG overnight measures, by breathing sound analysis during wakefulness [67]. Overall, combining different methodologies for wider reporting metrics, in addition to improved accuracy, may provide a more well-rounded, comprehensive screening tool for future use.

## Conclusions

Non-invasive detection during wakefulness of OSA is important as it can resolve many current major issues such as long waiting time to have an overnight PSG and lack of OSA diagnosis by reducing the need for PSG assessment through a quick and accurate screening during wakefulness, thus, significantly reducing the economic burden of OSA on healthcare. In addition, a reliable, comprehensive OSA detection tool would reduce possible perioperative morbidity and mortality, as well as facilitate faster treatment. There exist many studies that have investigated OSA screening during wakefulness, and yet, as suggested throughout the present review, opportunities for improvement exist to provide a measure for severity rather than only screening for OSA and non-OSA populations.

In this paper, different techniques for OSA detection during wakefulness are divided based on the main used methodology like imaging techniques, negative expiratory pressure, facial image landmarks, pharyngometry, breathing sound analysis, speech signal analysis, and questionnaires. For each technique, all related papers are reviewed and summarized to show the main outcome. This review also highlights the road map for the design specifications which are required or preferred in any feature methodology for the wakefulness technique of OSA detection.

The future open path for research in this area will be the design of more comfortable, reliable, and accurate devices to provide comfortable, cost-effective, and accurate ways for wakefulness detection of OSA and its severity; these will reduce the need for PSG recordings, especially for the initial screening. In a nutshell, this review shows that there is an increased focus by researchers on developing techniques for OSA detection during wakefulness. Although there are promising results from surveyed papers, there is a need for more clinical validation of these methods on larger populations.

## References

[1] Colten HR, Altevogt BM (2006) Sleep disorders and sleep deprivation: an unmet public health problem. The national academies press, Washington, D.C. 10.17226/11617 20669438

[2] Kushida CA, Littner MR, Morgenthaler T (2005). Practice parameters for the indications for polysomnography and related procedures: an update for 2005. Sleep.

[3] American Academy of Sleep Medicine (2005) International classification of sleep disorders: diagnostic & coding manual, 2nd ed. American Academy of Sleep Medicine, Westchester, IL

[4] Berry RB, Brooks R, Gamaldo CE (2012). The AASM manual for the scoring of sleep and associated events. Rules Terminology Techn Specifications Darien Illinois Am Acad Sleep Med.

[5] Bradley TD, Floras JS (2013). Sleep apnea: implications in cardiovascular and cerebrovascular disease.

[6] Butt M, Dwivedi G, Khair O, Lip GYH (2010). Obstructive sleep apnea and cardiovascular disease. Int J Cardiol.

[7] Espiritu JRD (2021) Health consequences of obstructive sleep apnea. In: Management of obstructive sleep apnea. Springer International Publishing, Cham, pp 23–43. 10.1007/978-3-030-54146-0_3

[8] Yoshihisa A, Takeishi Y (2019). Sleep disordered breathing and cardiovascular diseases. J Atheroscler Thromb.

[9] Noda A, Yasuma F, Miyata S (2019). Sleep fragmentation and risk of automobile accidents in patients with obstructive sleep apnea—sleep fragmentation and automobile accidents in OSA. Health N Hav.

[10] Young T, Finn L, Peppard PE (2008). Sleep disordered breathing and mortality: eighteen-year follow-up of the Wisconsin Sleep Cohort. Sleep.

[11] American Academy of Sleep Medicine (2016) Hidden health crisis costing america billions: underdiagnosing and undertreating obstructive sleep apnea draining healthcare system. Frost & Sullivan, Darien, IL

[12] The Harvard Medical School Division of Sleep Medicine (2010). The price of fatigue: the surprising economic costs of unmanaged sleep apnea.

[13] Tregear S, Reston J, Schoelles K, Phillips B (2009). Obstructive sleep apnea and risk of motor vehicle crash: systematic review and meta-analysis. J Clin Sleep Med.

[14] Stewart SA, Skomro R, Reid J (2015). Improvement in obstructive sleep apnea diagnosis and management wait times: a retrospective analysis of a home management pathway for obstructive sleep apnea. Can Respir J.

[15] Yadollahi A, Moussavi Z (2009) Acoustic obstructive sleep apnea detection. In: 2009 Annual international conference of the ieee engineering in medicine and biology society. IEEE, pp 7110–7113. 10.1109/IEMBS.2009.5332870 10.1109/IEMBS.2009.5332870 19963947

[16] Chen L, Pivetta B, Nagappa M (2021). Validation of the STOP-Bang questionnaire for screening of obstructive sleep apnea in the general population and commercial drivers: a systematic review and meta-analysis. Sleep Breath.

[17] Mazzotti DR, Keenan BT, Thorarinsdottir EH (2022). Is the Epworth Sleepiness Scale sufficient to identify the excessively sleepy subtype of OSA?. Chest.

[18] El-Sayed IH (2012). Comparison of four sleep questionnaires for screening obstructive sleep apnea. Egypt J Chest Dis Tuberc.

[19] Nagappa M, Liao P, Wong J (2015). Validation of the STOP-Bang questionnaire as a screening tool for obstructive sleep apnea among different populations: a systematic review and meta-analysis. PLoS One.

[20] American Society of Anesthesiologists (2014) Practice guidelines for the perioperative management of patients with obstructive sleep apnea: An updated report. Anesthesiology 120:268–286. 10.1097/ALN.0000000000000053 10.1097/ALN.0000000000000053 24346178

[21] Finkelstein Y, Wolf L, Nachmani A (2014). Velopharyngeal anatomy in patients with obstructive sleep apnea versus normal subjects. J Oral Maxillofac Surg.

[22] Lan Z, Itoi A, Takashima M (2006). Difference of pharyngeal morphology and mechanical property between OSAHS patients and normal subjects. Auris Nasus Larynx.

[23] Younes M (2008). Role of respiratory control mechanisms in the pathogenesis of obstructive sleep disorders. J Appl Physiol.

[24] Malhotra A, Pillar G, Fogel R (2001). Upper-airway collapsibility: measurements and sleep effects. Chest.

[25] Ryan CM (1985). Bradley TD (2005) Pathogenesis of obstructive sleep apnea. J Appl Physiol.

[26] Betts JG, DeSaix P, Johnson E et al (2022) Anatomy and physiology, 2nd ed. OpenStax, Houston, TX

[27] Elwali A, Moussavi Z (2016). Obstructive sleep apnea screening and airway structure characterization during wakefulness using tracheal breathing sounds. Ann Biomed Eng.

[28] Riley R, Guilleminault C, Herran J, Powell N (1983). Cephalometric analyses and flow-volume loops in obstructive sleep apnea patients. Sleep.

[29] Haponik EF, Smith PL, Bohlman ME (1983). Computerized tomography in obstructive sleep apnea. Correlation of airway size with physiology during sleep and wakefulness. Am Rev Respir Dis.

[30] Schwab RJ, Gefter WB, Hoffman EA (1993). Dynamic upper airway imaging during awake respiration in normal subjects and patients with sleep disordered breathing. Am Rev Respir Dis.

[31] Ciscar MA, Juan G, Martõ Ânez V (2001). Magnetic resonance imaging of the pharynx in OSA patients and healthy subjects. Eur Respir J.

[32] Molnár V, Molnár A, Lakner Z (2023). The prognostic role of ultrasound and magnetic resonance imaging in obstructive sleep apnoea based on lateral oropharyngeal wall obstruction. Sleep Breath.

[33] Bindi I, Ori M, Marchegiani M (2022). Diagnosis of upper airways collapse in moderate-to-severe OSAHS patients: a comparison between drug-induced sleep endoscopy and the awake examination. Eur Arch Otorhinolaryngol.

[34] Molnár V, Lakner Z, Molnár A (2022). Ultrasound and magnetic resonance imaging of the tongue in obstructive sleep apnoea. Appl Sci.

[35] Molnár V, Lakner Z, Molnár A (2022). The predictive role of subcutaneous adipose tissue in the pathogenesis of obstructive sleep apnoea. Life.

[36] Darquenne C, Elliott AR, Sibille B (2018). Upper airway dynamic imaging during tidal breathing in awake and asleep subjects with obstructive sleep apnea and healthy controls. Physiol Rep.

[37] Schwab RJ, Pasirstein M, Pierson R (2003). Identification of upper airway anatomic risk factors for obstructive sleep apnea with volumetric magnetic resonance imaging. Am J Respir Crit Care Med.

[38] Bland JM, Altman DG (2000). The odds ratio. BMJ.

[39] Kim TY, Son J, Kim KG (2011). The recent progress in quantitative medical image analysis for computer aided diagnosis systems. Healthc Inform Res.

[40] Lahav Y, Rosenzweig E, Heyman Z (2009). Tongue base ultrasound: a diagnostic tool for predicting obstructive sleep apnea. Ann Otol Rhinol Laryngol.

[41] Tamisier R, Wuyam B, Nicolle I (2005). Awake flow limitation with negative expiratory pressure in sleep disordered breathing. Sleep Med.

[42] Sanders MH, Martin RJ, Pennock BE, Rogers RM (1981). The detection of sleep apnea in the awake patient: the “saw-tooth” sign. JAMA.

[43] Carrera HL, Marcus CL, McDonough JM (2015). Negative expiratory pressure technique: an awake test to measure upper airway collapsibility in adolescents. Sleep.

[44] Romano S, Salvaggio A, Hirata RP (2011). Upper airway collapsibility evaluated by a negative expiratory pressure test in severe obstructive sleep apnea. Clinics.

[45] Romano S, Salvaggio A, Lo BA (2011). A negative expiratory pressure test during wakefulness for evaluating the risk of obstructive sleep apnea in patients referred for sleep studies. Clinics (Sao Paulo).

[46] Lim J, Alshaer H, Ghahjaverestan NM, Bradley TD (2023). Relationship between airflow limitation in response to upper airway negative pressure during wakefulness and obstructive sleep apnea severity. Sleep Breath.

[47] Gao J, Yang Y, Lin P, Park DS (2018). Computer vision in healthcare applications. J Healthc Eng.

[48] Siddiqi MH, Khan K, Khan RU, Alsirhani A (2022). Face image analysis using machine learning: a survey on recent trends and applications. Electronics.

[49] Okubo M, Suzuki M, Horiuchi A (2006). Morphologic analyses of mandible and upper airway soft tissue by MRI of patients with obstructive sleep apnea hypopnea syndrome. Sleep.

[50] Miles PG, Vig PS, Weyant RJ (1996). Craniofacial structure and obstructive sleep apnea syndrome–a qualitative analysis and meta-analysis of the literature. Am J Orthod Dentofacial Orthop.

[51] Espinoza-Cuadros F, Fernández-Pozo R, Toledano DT (2015). Speech signal and facial image processing for obstructive sleep apnea assessment. Comput Math Methods Med.

[52] Tyan M, Espinoza-Cuadros F, Pozo RF (2017). Obstructive sleep apnea in women: study of speech and craniofacial characteristics. JMIR Mhealth Uhealth.

[53] Lee RWW, Petocz P, Prvan T (2009). Prediction of obstructive sleep apnea with craniofacial photographic analysis. Sleep.

[54] Balaei AT, Sutherland K, Cistulli PA, de Chazal P (2017) Automatic detection of obstructive sleep apnea using facial images. In: 2017 IEEE 14th International symposium on biomedical imaging (ISBI 2017). IEEE, pp 215–218. 10.1109/ISBI.2017.7950504

[55] Sutherland K, Lee RWW, Petocz P (2016). Craniofacial phenotyping for prediction of obstructive sleep apnoea in a Chinese population. Respirology.

[56] Monna F, Ben Messaoud R, Navarro N (2022). Machine learning and geometric morphometrics to predict obstructive sleep apnea from 3D craniofacial scans. Sleep Med.

[57] Ciaparrone G, Chiariglione L, Tagliaferri R (2022). A comparison of deep learning models for end-to-end face-based video retrieval in unconstrained videos. Neural Comput Appl.

[58] Islam SMS, Mahmood H, Al-Jumaily AA, Claxton S (2018) Deep learning of facial depth maps for obstructive sleep apnea prediction. In: 2018 International conference on machine learning and data engineering (iCMLDE). IEEE, pp 154–157.10.1109/iCMLDE.2018.00036

[59] Dae GJ, Hae YC, Grunstein RR, Yee B (2004). Predictive value of Kushida index and acoustic pharyngometry for the evaluation of upper airway in subjects with or without obstructive sleep apnea. J Korean Med Sci.

[60] Kushida CA, Efron B, Guilleminault C (1997). A predictive morphometric model for the obstructive sleep apnea syndrome. Ann Intern Med.

[61] Gelardi M, Del Giudice AM, Cariti F (2007). Acoustic pharyngometry: clinical and instrumental correlations in sleep disorders. Braz J Otorhinolaryngol.

[62] DeYoung PN, Bakker JP, Sands SA (2013). Acoustic pharyngometry measurement of minimal cross-sectional airway area is a significant independent predictor of moderate-to-severe obstructive sleep apnea. J Clin Sleep Med.

[63] Shivarov G (2022). Anthropometry and acoustic pharyngometry of the oral cavity in sleep-disordered breathing. J IMAB – Ann Proc Sci Pap.

[64] Salisbury JI, Sun Y (2007). Rapid screening test for sleep apnea using a nonlinear and nonstationary signal processing technique. Med Eng Phys.

[65] Simply RM, Dafna E, Zigel Y (2018) Obstructive sleep apnea (OSA) classification using analysis of breathing sounds during speech. In: 2018 26th European signal processing conference (EUSIPCO). IEEE, pp 1132–1136. 10.23919/EUSIPCO.2018.8553353

[66] Moussavi Z, Elwali A, University of Manitoba (2021) System and methods for screening obstructive sleep apnea during wakefulness using anthropometric information and tracheal breathing sounds. U.S. Patent Application 17/349,298.

[67] Elwali A, Moussavi Z (2021). Predicting polysomnography parameters from anthropometric features and breathing sounds recorded during wakefulness. Diagnostics.

[68] Hajipour F, Moussavi Z (2019) Spectral and higher order statistical characteristics of expiratory tracheal breathing sounds during wakefulness and sleep in people with different levels of obstructive sleep apnea. J Med Biol Eng 39:244–250.10.1007/s40846-018-0409-7

[69] Elwali A, Moussavi Z (2019). Determining breathing sound features representative of obstructive sleep apnea during wakefulness with least sensitivity to other risk factors. J Med Biol Eng.

[70] Elwali A, Moussavi Z (2019) A novel decision making procedure during wakefulness for screening obstructive sleep apnea using anthropometric information and tracheal breathing sounds. Sci Rep 9:11467. 10.1038/s41598-019-47998-5 10.1038/s41598-019-47998-5 PMC6685971 31391528

[71] Montazeri A, Giannouli E, Moussavi Z (2012). Assessment of obstructive sleep apnea and its severity during wakefulness. Ann Biomed Eng.

[72] Hajipour F, Jozani MJ, Moussavi Z (2020). A comparison of regularized logistic regression and random forest machine learning models for daytime diagnosis of obstructive sleep apnea. Med Biol Eng Comput.

[73] Elwali A, Meza-Vargas S, Moussavi Z (2019). Using tracheal breathing sounds and anthropometric information for screening obstructive sleep apnoea during wakefulness. J Med Eng Technol.

[74] Hajipour F, Jozani MJ, Elwali A, Moussavi Z (2019). Regularized logistic regression for obstructive sleep apnea screening during wakefulness using daytime tracheal breathing sounds and anthropometric information. Med Biol Eng Comput.

[75] Pasterkamp H (1996). Posture-dependent change of tracheal sounds at standardized flows in patients with obstructive sleep apnea. Chest J.

[76] Sola-Soler J, Fiz JA, Torres A, Jane R (2014) Identification of obstructive sleep apnea patients from tracheal breath sound analysis during wakefulness in polysomnographic studies. In: 2014 36th Annual international conference of the ieee engineering in medicine and biology society. IEEE, pp 4232–4235. 10.1109/embc.2014.6944558 10.1109/EMBC.2014.6944558 25570926

[77] Sola-Soler J, Jane R, Fiz JA, Morera J (2008) Formant frequencies of normal breath sounds of snorers may indicate the risk of obstructive sleep apnea syndrome. In: 2008 30th Annual international conference of the IEEE engineering in medicine and biology society. IEEE, pp 3500–3503. 10.1109/IEMBS.2008.4649960 10.1109/IEMBS.2008.4649960 19163463

[78] Pozo RF, Murillo JLB, Gmez LH (2009). Assessment of severe apnoea through voice analysis, automatic speech, and speaker recognition techniques. EURASIP J Adv Sig Process.

[79] Montero Benavides A, Fernández Pozo R, Toledano DT (2014). Analysis of voice features related to obstructive sleep apnoea and their application in diagnosis support. Comput Speech Lang.

[80] Espinoza-Cuadros F, Fernández-Pozo R, Toledano DT (2016). Reviewing the connection between speech and obstructive sleep apnea. Biomed Eng Online.

[81] Montero Benavides A, Blanco Murillo JL, Fernández Pozo R (2016). Formant frequencies and bandwidths in relation to clinical variables in an obstructive sleep apnea population. J Voice.

[82] Goldshtein E, Tarasiuk A, Zigel Y (2011). Automatic detection of obstructive sleep apnea using speech signals. IEEE Trans Biomed Eng.

[83] Simply RM, Dafna E, Zigel Y (2020). Diagnosis of obstructive sleep apnea using speech signals from awake subjects. IEEE J Sel Top Sign Proces.

[84] Solé-Casals J, Munteanu C, Martín OC (2014). Detection of severe obstructive sleep apnea through voice analysis. Appl Soft Comput.

[85] Pang K-G, Hsung T-C, Liao G (2022). Obstructive sleep apnea detection using speech signals with high frequency components. J Commun.

[86] Yılmaz D, Yıldız M, Uyar Toprak Y, Yetkin S (2023). Obstructive sleep apnea detection with nonlinear analysis of speech. Biomed Signal Process Control.

[87] Ramachandran SK, Josephs LA (2009). A meta-analysis of clinical screening tests for obstructive sleep apnea. Anesthesiology.

[88] Behar JA, Palmius N, Daly J (2017). Sleep questionnaires in screening for obstructive sleep apnoea. Comput Cardiol (2010).

[89] Kump K, Whalen C, Tishler PV (1994). Assessment of the validity and utility of a sleep-symptom questionnaire. Am J Respir Crit Care Med.

[90] Pataka A, Daskalopoulou E, Kalamaras G (2014). Evaluation of five different questionnaires for assessing sleep apnea syndrome in a sleep clinic. Sleep Med.

[91] Solecka Š, Matler K, Kostlivý T (2022). A comparison of the reliability of five sleep questionnaires for the detection of obstructive sleep apnea. Life.

[92] Shahar E, Whitney CW, Redline S (2001). Sleep-disordered breathing and cardiovascular disease: cross-sectional results of the Sleep Heart Health Study. Am J Respir Crit Care Med.

[93] Caseiro P, Fonseca-Pinto R, Andrade A (2010). Screening of obstructive sleep apnea using Hilbert-Huang decomposition of oronasal airway pressure recordings. Med Eng Phys.

[94] Young T, Palta M, Dempsey J (1993). The occurrence of sleep-disordered breathing among middle-aged adults. N Engl J Med.

[95] Simas R, Maestri F, Normando D (2014). Controlling false positive rates in research and its clinical implications. Dental Press J Orthod.

[96] Olliaro P, Torreele E (2021). Managing the risks of making the wrong diagnosis: first, do no harm. Int J Infect Dis.

[97] Burke JF, Sussman JB, Kent DM, Hayward RA (2015). Three simple rules to ensure reasonably credible subgroup analyses. The BMJ.

[98] Kim HE, Kim HH, Han BK (2020). Changes in cancer detection and false-positive recall in mammography using artificial intelligence: a retrospective, multireader study. Lancet Digit Health.

[99] Meshkinfamfard S, Gorban A, Tyukin I (2018) Tackling rare false-positives in face recognition: a case study. In: 2018 IEEE 20th International conference on high performance computing and communications; IEEE 16th International conference on smart city; IEEE 4th International conference on data science and systems (HPCC/SmartCity/DSS). IEEE, pp 1592–1598. 10.1109/HPCC/SMARTCITY/DSS.2018.00260

[100] Shen Y, Shamout FE, Oliver JR (2021). Artificial intelligence system reduces false-positive findings in the interpretation of breast ultrasound exams. Nat Commun.

[101] Alsubie HS, BaHammam AS (2017). Obstructive sleep apnoea: children are not little adults. Paediatr Respir Rev.

